# Biomimetic Hierarchical Construction of Anti‐Tumor Polyoxopalladates for Cancer Therapy

**DOI:** 10.1002/anie.202505564

**Published:** 2025-04-14

**Authors:** Yue Zhao, Zheran Liu, Zijian Qin, Qinlong Wen, Jing Du, Xiang‐Yu Ren, Chao‐Qin Chen, Xingchen Peng, Ulrich Kortz, Peng Yang

**Affiliations:** ^1^ College of Chemistry and Chemical Engineering Hunan University Changsha 410082 P.R. China; ^2^ Department of Biotherapy Cancer Center West China Hospital Sichuan University Chengdu 610041 P.R. China; ^3^ School of Science Constructor University Campus Ring 1, 28759 Bremen Germany; ^4^ Testing and Analysis Center Hebei Normal University Shijiazhuang 050024 P.R. China

**Keywords:** Biomimetic synthesis, Hierarchical assembly, Lacunary precursor, Polyoxometalate, Polyoxopalladate

## Abstract

Inspired by the construction scheme of biomacromolecules, a hierarchical assembly based on the lacunary polyoxopalladate (POP) of [SrPd_12_O_6_(OH)_3_(PhAsO_3_)_6_(OAc)_3_]^4−^ (SrPd_12_) has been achieved. As a structurally programmable molecular building block, SrPd_12_ is used to evolve from monomer via dimer to supramolecular aggregates in a controlled manner. In such process, the open‐shell‐type monomers are covalently integrated into bowl‐ or cage‐like dimers via a direct or indirect splicing strategy. Upon that, hydrogen bond and hydrophobic effects are further hired to fabricate supramolecular aggregates of varied host–guest archetypes, thereby completing a hierarchical construction. In consideration of the combined advantages of noble metals and polyoxometalates in cancer treatment, both in vitro and in vivo anti‐tumor assays of these SrPd_12_‐derived POPs were studied in detail. A structure‐dependent anti‐tumor activitywas observed, originating from an imbalance of damage and repair of DNA as anti‐tumor mechanism.

## Graphical abstract

Engineering of lacunary polyoxopalladates resulted in a hierarchical assembly that resembles the construction of biomacromolecules. An ordered structural evolution from monomer via dimer to supramolecular aggregates was achieved by cooperative covalent and supramolecular bonding effects, resulting in enhanced anti‐tumor activity via DNA damage.

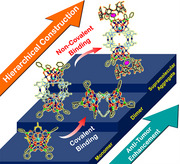

## Introduction

Learning from the construction scheme of biomacromolecules provides chemists with new ideas and solutions that are rich in natural wisdom.^[^ ^1^, ^2^, ^3^ ^]^ However, such a learning process is difficult, as advanced synthetic skills are required for emulating the sophisticated structure of biomacromolecules.^[^ ^4^, ^5^ ^]^ From amino acid via polypeptide to protein, this course of fabrication typically experiences a hierarchical assembly. That is cascading monomers to oligo‐/polymers followed by their supramolecular organization. Of this, the teamwork of covalent bonding (e.g., amide bond) and intermolecular weak forces (e.g., hydrogen bond and hydrophobic effect) is vital, while the overall management of them is still challenging.^[^ ^6^ ^]^ In response to this, the development of molecular building blocks (MBBs) with well‐defined reactive sites for both covalent and non‐covalent interactions represents the research focus of biomimetic synthesis.^[^ ^7^, ^8^ ^]^

As a category of classic MBBs, the outstanding advantage of polyoxometalates (POMs) lies in the tunability of structure and composition.^[^ ^9^, ^10^, ^11^ ^]^ On the one hand, a varied number of addenda atoms are allowed to be selectively removed, resulting in lacunary POMs available as synthetic precursors.^[^ ^12^ ^]^ The freed space and enhanced nucleophilicity enable them to coordinate electrophiles of different types, yielding multimeric species with nanometer scale.^[^ ^13^ ^]^ On the other hand, a part of surface oxo groups of POMs could be replaced by organic ligands (e.g., polycarboxylic acids and polyhydric alcohols), where the peripheral functions are able to target electrophiles in particular directions, or to support intermolecular interactions at specific locations.^[^ ^14^, ^15^, ^16^ ^]^ For example, organic–inorganic hybrid POMs of Anderson–Evans and Lindqvist‐type topologies have been widely employed as synthons in making porous materials and supramolecular aggregates.^[^ ^17^, ^18^ ^]^ In this context, it would be of great interest to integrate the above two strategies, and consequently to prepare organically modified lacunary POMs (OL‐POMs). The co‐reinforcement of assembling capacity and machining precision is expected to significantly promote their availability and controllability as MBBs in hierarchical construction. Nevertheless, synthesis of OL‐POMs has hitherto met with limited success, possibly because of the strong electrostatic repulsion between the negatively charged metal‐oxo frameworks and deprotonated ligands.^[^ ^19^, ^20^ ^]^ Moreover, most of the reported OL‐POMs are formed in situ and frequently difficult to be reprocessed, which severely impedes their spread and application in hierarchical synthesis.^[^ ^12^ ^]^

Lately, the emergence of polyoxo‐noble‐metalates delivers new opportunities to access OL‐POMs.^[^ ^21^, ^22^, ^23^, ^24^ ^]^ In particular, polyoxopalladates (POPs) stand out, highlighted by the designability in both structure and function.^[^ ^25^ ^]^ By means of basification (9 ≤ pH ≤ 11), a certain amount of square‐planar {PdO_4_} units could depart from the cubic dodecanuclear matrix, resulting in mono‐ and di‐vacant species.^[^ ^26^, ^27^ ^]^ Meanwhile, different from the embedded one or two heterogroups in conventional Keggin or Wells–Dawson‐type POMs, a large number of heterogroups are placed outside of POPs. The exposed and well‐distributed organic functions are apt to bridge the neighboring molecules, thereby yielding POP‐based polymeric materials.^[^ ^28^ ^]^ Among the reported POPs, the acetate‐functionalized, tri‐lacunary [SrPd_12_O_6_(OH)_3_(PhAsO_3_)_6_(OAc)_3_]^4−^ (**SrPd_12_**) has provoked our attention.^[^ ^29^ ^]^ Broken from the star‐shaped pentadecanuclear matrix, **SrPd_12_** might serve as an ideal MBB for hierarchical assembly in consideration of its built‐in advantages: i) open‐shell archetype with less steric hindrance; ii) labile acetates available for ligand exchange; iii) enriched aromatic groups for intermolecular bindings; iv) rigid scaffold consolidated by Sr^II^ template; and v) excellent stability in both aqueous and non‐aqueous solutions. In addition to the structural interests, **SrPd_12_** inherits the bioactivity of noble metal‐based pharmaceuticals such as cisplatin.^[^ ^30^ ^]^ The considerable anti‐tumor activities against various cancer cells might be orchestrated by a set of variables involving shape, size, and charge of POPs.^[^ ^31^, ^32^, ^33^, ^34^, ^35^ ^]^

**Scheme 1:**
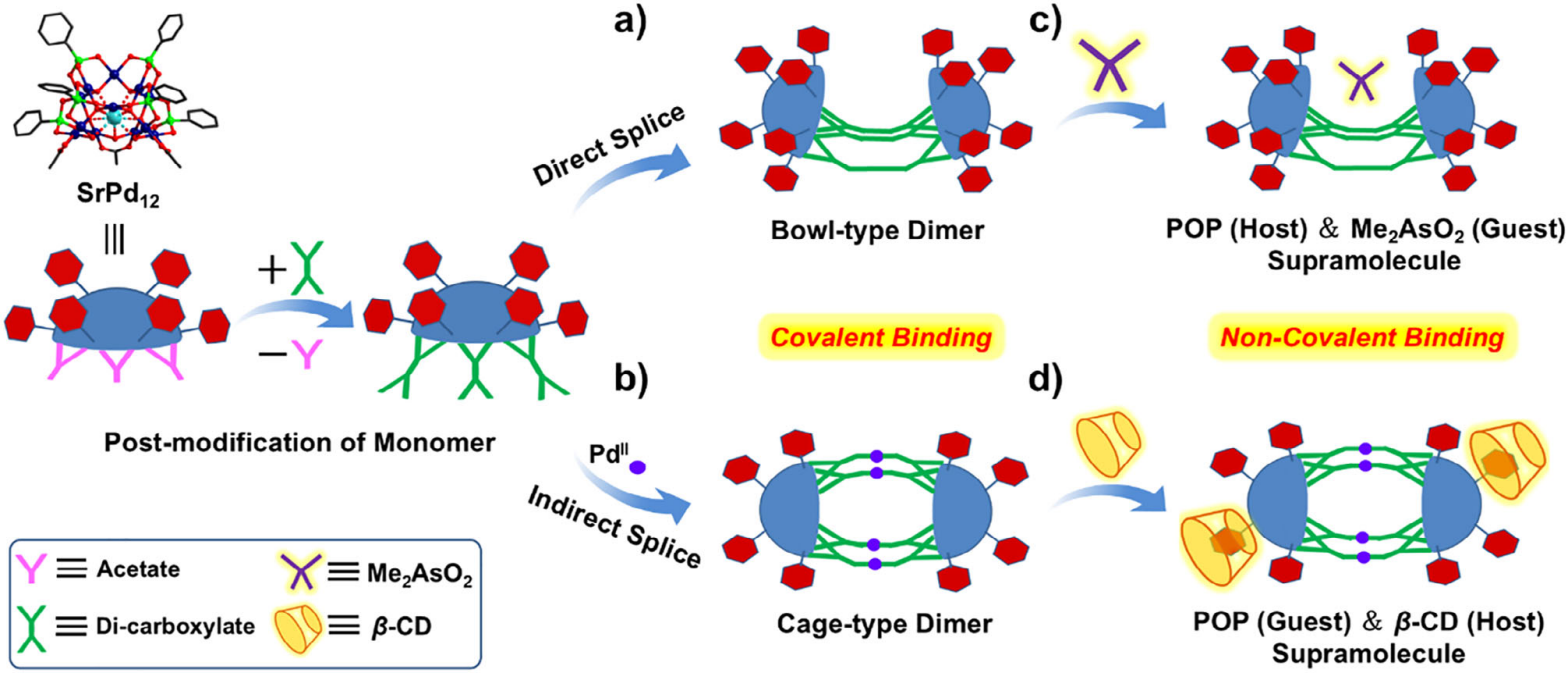
Biomimetic hierarchical construction of **SrPd_12_** as MBB via covalently direct or indirect splice into bowl‐type dimers a,b) and non‐covalently supramolecular aggregation into varied host–guest assemblies c,d).

Given the advantage of structural editability, the hierarchical construction of **SrPd_12_** is worth exploring, which would help to popularize the biomimetic synthetic technique in POM chemistry. Meanwhile, the anti‐tumor potential of **SrPd_12_** might be further unleashed upon structural modification, followed by the improved understanding of anti‐tumor mechanism. Inspired by these, herein, **SrPd_12_** has been revisited via a post‐synthetic modification approach. The di‐carboxylate substituted monomeric **SrPd_12_** could be spliced into dimers of bowl‐ or cage‐like configurations via a direct (Scheme 1a) or indirect method (Scheme 1b), respectively. On top of this, additional molecules such as dimethylarsinate ({Me_2_AsO_2_}) and β‐cyclodextrin (β‐CD) participated respectively as guest (Scheme 1c) and host (Scheme 1d) component to complete distinct host–guest assemblies. Assisted by the joint efforts of covalent and non‐covalent bindings, customized evolution of molecular structure as well as biological function has been achieved, in a manner similar to the construction of proteins evolved in nature.

## Results and Discussion

### Post‐Modification of Open‐Shell‐Type Monomer

**Figure 1:**
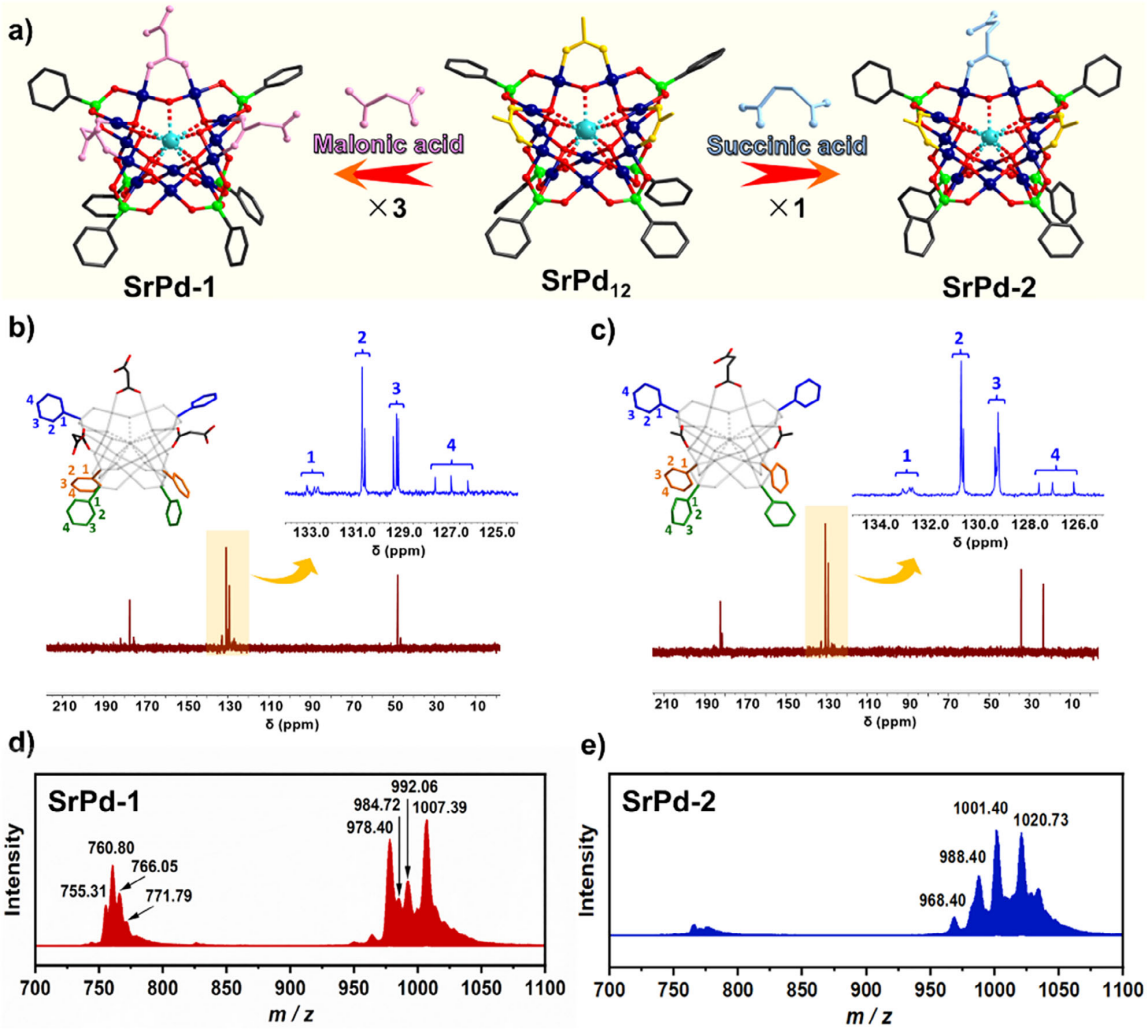
a) Syntheses of **SrPd‐1** and **SrPd‐2** by post‐modification of **SrPd_12_** precursor. Color code: acetate, yellow; Pd, dark blue; As, green; Sr, turquoise; C, gray; O, red. b),c) ^13^C NMR spectra of **SrPd‐1** and **SrPd‐2** (100 mM, pH = 7.0, D_2_O, 298 K). d),e) Negative‐ion mass spectra of **SrPd‐1** and **SrPd‐2** (2.0 × 10^−2^ mM, H_2_O:CH_3_CN = 9:1, 298 K).

To date, the cuboid [MPd_12_O_8_(LXO_3_)_8_]^n−^ and star‐like [MPd_15_O_10_(LXO_3_)_10_]^n−^ (M = guest metal; LXO_3_ = heterogroup, X = As^V^, P^V^, etc., L = O or organic function) represent the most prevalent structural models of POPs (Figure S1).^[^ ^22^, ^36^ ^]^ With regards to the newly emerged open‐shell archetype, the formation of **SrPd_12_** significantly relies on the presence of Sr^II^ as template.^[^ ^29^ ^]^ Associated with a unique energy state, three Pd^II^ addenda and four {PhAsO_3_} heterogroups are removed from the assumed star‐type [SrPd_15_O_10_(PhAsO_3_)_10_]^8−^ matrix, yielding the tri‐lacunary scaffold of **SrPd_12_** (Figure S2).^[^ ^37^ ^]^ Of prime interest, the three labile acetate groups in **SrPd_12_** are available to be substituted by other molecules such as H_2_O, which has been validated by both theoretical and experimental studies.^[^ ^29^, ^38^ ^]^ With this in mind, two kinds of aliphatic diacids (malonic acid and succinic acid) were hired to replace the acetates in **SrPd_12_**, resulting in two post‐modified POPs of [SrPd_12_O_6_(OH)_3_(PhAsO_3_)_6_(C_3_H_2_O_4_)_3_]^7−^ (**SrPd‐1**) and [SrPd_12_O_6_(OH)_3_(PhAsO_3_)_6_(OAc)_2_(C_4_H_4_O_4_)]^5−^ (**SrPd‐2**) (Figure 1a).^[^ ^39^ ^]^ These POPs were prepared by a straightforward one‐pot approach and isolated as alkali metal salts in high yield (>70%).

Obviously, the Pd‐oxo framework of **SrPd_12_** has been well preserved in the derived species. For **SrPd‐1**, three propanedioate molecules take the positions that originally belonged to acetates. By contrast, only the acetate located at the plane of symmetry has been substituted by a succinate ligand in **SrPd‐2**. Attempts to prepare the completely tri‐succinate‐substituted POP have been carried out but without success. This might be attributed to the repulsion among the aliphatic carboxylate arms that are negatively charged and sterically demanding. No absorption bands centered at ∼1700 cm^−1^ have been observed in the FT‐IR spectra of **SrPd‐1** and **SrPd‐2**, indicating the attached diacids are fully deprotonated (Figure S3).^[^ ^40^ ^]^ To examine their stability in both solution and gas phases, characterizations of NMR and ESI‐MS have been performed (Figure S4). According to the *C*_s_ point group of symmetry, six {PhAsO_3_} heterogroups in the open‐shell structure can be categorized into three subsets. As depicted in Figure 1b,c, ^13^C NMR signals of magnetically inequivalent aromatic carbon atoms could be unambiguously identified, indicating the structure of both POPs are intact in water. In terms of ESI‐MS study, the main envelopes situated at *m*/*z* = 755.31, 760.80, 766.05, and 771.79 are assigned to a series of −4 charged protonated and sodium form of **SrPd‐1** with formulas of {H_3_ **SrPd‐1**}^4−^, {H_2_Na**SrPd‐1**}^4−^, {HNa_2_ **SrPd‐1**}^4−^, and {Na_3_ **SrPd‐1**}^4−^, respectively (Figure 1d). For **SrPd‐2**, the ionic moieties observed at *m*/*z* = 988.40, 1001.40 and 1020.73 correspond to the −3 charged species of {H_2_ **SrPd‐2**(H_2_O)}^3−^, {KH**SrPd‐2**(H_2_O)}^3−^, and {KNa**SrPd‐2**(H_2_O)_3_}^3−^ (Figure 1e). In agreement with the NMR results, the above ESI‐MS data strongly support the prominent solution stability of **SrPd‐1** and **SrPd‐2**, as most peaks correspond to the intact polyanions. Meanwhile, similar to the parent **SrPd_12_**, the dynamic nature of both POPs has been noticed as well. For example, fragments of [H_2_SrPd_12_O_6_(OH)_3_(C_6_H_5_AsO_3_)_6_(C_3_H_2_O_4_)_2_(H_2_O)]^3−^ (*m*/*z* = 978.40) and [HSrPd_12_O_6_(OH)_3_(C_6_H_5_AsO_3_)_6_(OAc)(C_4_H_4_O_4_)(H_2_O)]^3−^ (*m*/*z* = 968.40) have been received, corresponding to **SrPd‐1** and **SrPd‐2** with one propanedioate or acetate ligand being lost, respectively. Additional MS assignments for the major peaks are summarized in Table S1.

### Direct Splice of Bowl‐Type Dimer as Host

**Figure 2:**
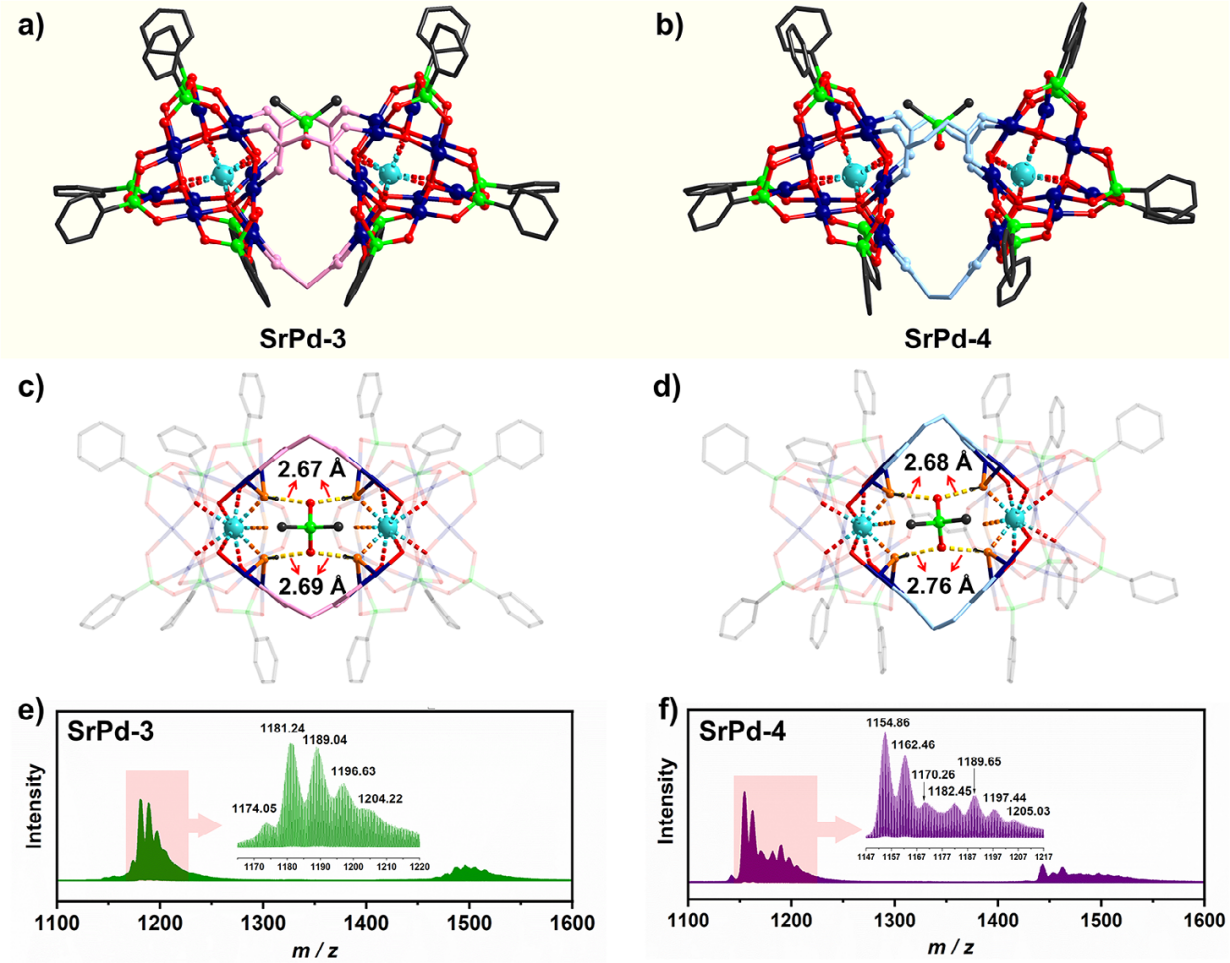
a),b) Structural representation of the bowl‐shaped **SrPd‐3** and **SrPd‐4** of anion@anion host‐guest archetype. c),d) {Me_2_AsO_2_} guest stabilized by hydrogen bonds (hydroxyl groups are marked by orange color). e),f) Negative‐ion mass spectra of **SrPd‐3** and **SrPd‐4** (2.0 × 10^−2^ mM, H_2_O:CH_3_CN = 9:1, 298 K).

In view of the idle carboxylate groups hanging outside, direct oligo‐/polymerization of such monomeric **SrPd‐1** and **SrPd‐2** can be envisaged. Upon increasing the reaction pH above 7.0, the corresponding dimers of [(Me_2_AsO_2_)⊂{SrPd_12_O_6_(OH)_3_(PhAsO_3_)_6_}_2_(C_3_H_2_O_4_)_3_]^9−^ (**SrPd‐3**) and [(Me_2_AsO_2_)⊂{SrPd_12_O_6_(OH)_3_(PhAsO_3_)_6_}_2_(C_4_H_4_O_4_)_3_]^9−^ (**SrPd‐4**) were obtained successfully. It is worth noting that dimethylarsinate {Me_2_AsO_2_} has to take part in the assembly process, serving as guest molecule nested in the POP host. For the host, a couple of acetate‐free **SrPd_12_** are bridged by three di‐carboxylate linkers, displaying a bowl‐shaped conformation with *C*_2_ *_v_* symmetry (Figure 2a,b). The Pd‐oxo clusters at both ends are slightly upturned, which cooperate with two ligands to constitute the rim of the bowl, together with the remaining linker staying at the bottom. Thus, it can be seen from **SrPd‐4** that the full replacement of the three acetates in **SrPd_12_** by succinates is indeed possible, which might be attributed to the enhanced rigidity of the dimeric scaffold. On the other side, one {Me_2_AsO_2_} guest has been incorporated into the POP host, locating at the mouth of the half‐open cavity (Figure S5). It has been well documented that POPs are good at capturing metallic cations across the periodic table, yielding a vast array of host‐guest assemblies.^[^ ^25^, ^41^, ^42^, ^43^, ^44^ ^]^ For the first time, an anionic guest (Me_2_AsO_2_^−^) has been implanted into an anionic POP host successfully, which enriches further the type of host–guest interactions in POP chemistry. Such kind of combination (anion@anion) is rather difficult, mainly because of the strong electrostatic repulsion between both host and guest components. Without coordination binding, herein, hydrogen bonds are responsible for stabilizing the host–guest system. Two of the three hydroxyl functions around every {SrO_6_(OH)_3_} center interact with one oxygen atom of {Me_2_AsO_2_} each, resulting in four groups of hydrogen bonds with an average distance of 2.68 Å (**SrPd‐3**, Figure 2c) and 2.72 Å (**SrPd‐4**, Figure 2d), respectively. Substitutions of the guest molecule by other tetrahedral inorganic or hybrid oxyanions (e.g., MnO_4_^−^, CrO_4_^2−^, and Ph_2_PO_2_^−^) were unsuccessful, signifying the exclusive role of {Me_2_AsO_2_} in the construction of as‐made dimers. Regarding the solution behaviors, the structures of **SrPd‐3** and **SrPd‐4** are stable in aqueous solution as demonstrated by NMR characterizations (Figures S6 and S7). Meanwhile, the envelopes belonging to the intact POPs of host–guest archetype have also been identified clearly in their mass spectra (Figure 2e,f and Table S1). Interestingly, ionic moieties located at *m*/*z* = 1154.86, 1162.46 and 1170.26 can be formulated as {H_3_[SrPd_12_O_6_(OH)_3_(PhAsO_3_)_6_]_2_(C_4_H_4_O_4_)_3_}^5−^, {KH_2_[SrPd_12_O_6_(OH)_3_(PhAsO_3_)_6_]_2_(C_4_H_4_O_4_)_3_}^5−^ and {K_2_H[SrPd_12_O_6_(OH)_3_(PhAsO_3_)_6_]_2_(C_4_H_4_O_4_)_3_}^5−^, which come from the derivatives of **SrPd‐4** without any guest. These findings manifest that, as compared with **SrPd‐3**, {Me_2_AsO_2_} stands a chance to escape from the pocket of **SrPd‐4**, which might be ascribed to the larger and twisted cavity fenced by the more flexible succinates.

### Indirect Splice of Cage‐Type Dimer as Guest

**Figure 3:**
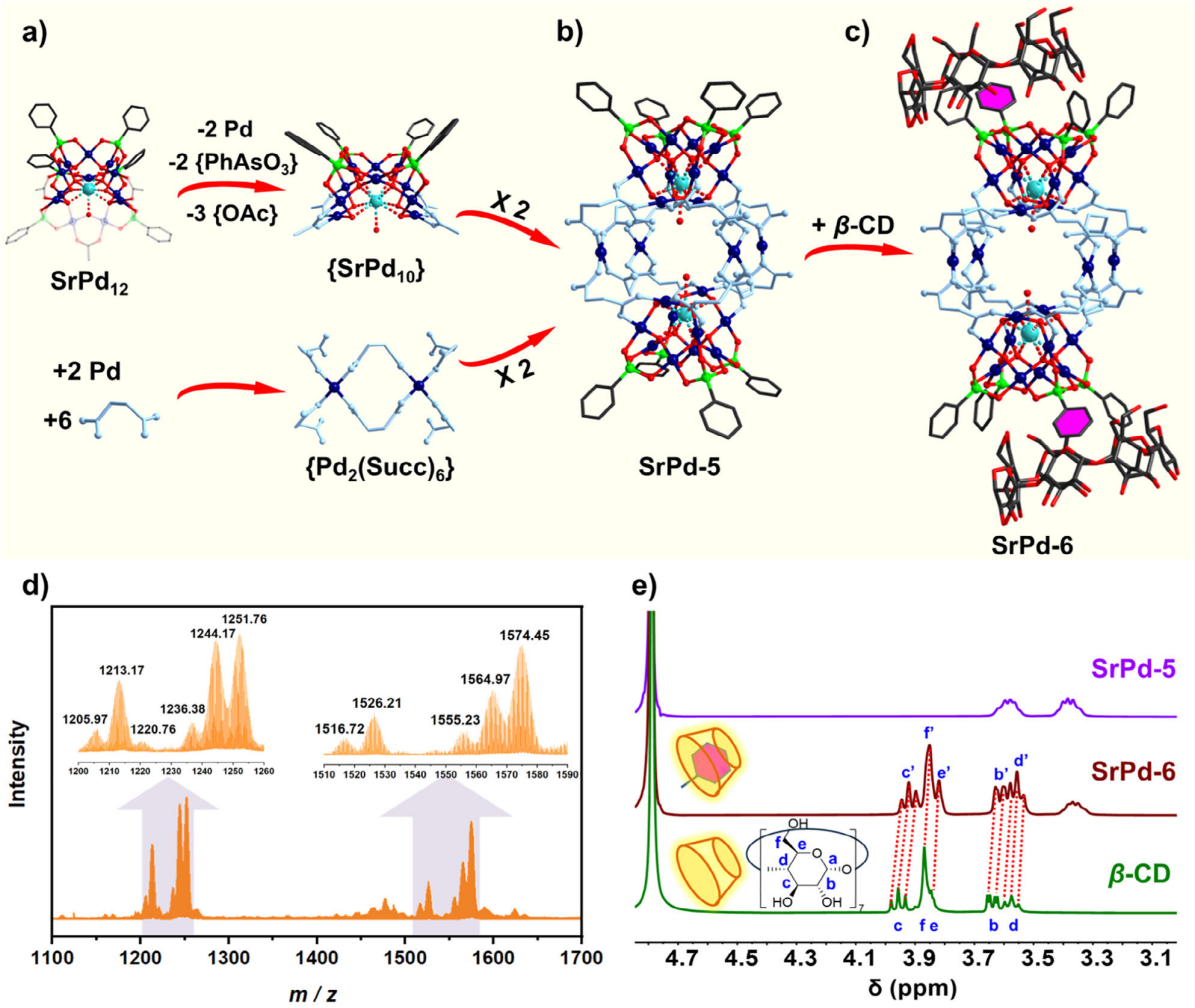
a) Formation path of {SrPd_10_} and {Pd_2_(Succ)_6_} fragments. b) Structural representation of the cage‐like **SrPd‐5**. c) Host–guest architecture of **SrPd‐6** constituted by β‐CD and **SrPd‐5**. d) Negative‐ion mass spectrum of **SrPd‐5** (2.0 × 10^−2^ mM, H_2_O:CH_3_CN = 9:1, 298 K). e) Chemical shift of ^1^H NMR signals belonging to β‐CD in **SrPd‐6** (20 mM, pH = 7.0, D_2_O, 298 K).

Beyond the straightforward dimerization stated above, additional Pd^II^ ions were added into the reaction system as transmitter for an indirect combination. Correspondingly, [{SrPd_10_O_4_(OH)_4_(H_2_O)(PhAsO_3_)_4_}_2_Pd_4_(C_4_H_4_O_4_)_12_]^12−^ (**SrPd‐5**) with an elongated cuboid configuration was obtained. Surprisingly, an unprecedented {SrPd_10_O_4_(OH)_4_(H_2_O)(PhAsO_3_)_4_} ({SrPd_10_}) has been evolved from **SrPd_12_** by losing two pairs of Pd^II^ addenda and {PhAsO_3_} heterogroups as well as three acetates (Figure 3a). It is noteworthy that the capture and stabilization of lacunary POPs is still difficult, and {SrPd_10_} represents the first example of penta‐vacant species in POP chemistry.^[^ ^12^ ^]^ The unsaturated coordination sites of Pd^II^ are then occupied by carboxylate groups from four succinate ligands. Meanwhile, a combination of two Pd^II^ and six succinates has afforded a {Pd_2_(C_4_H_4_O_4_)_6_} ({Pd_2_(Succ)_6_}) unit. Of this, all of the carboxylate functions adopt mono‐dentate mode to satisfy the square–planar geometry of Pd^II^. A pair of {Pd_2_(Succ)_6_} units occupy the equatorial zone and link two {SrPd_10_} fragments at both ends, forming a hollow chamber with the size of ca. 9.1 × 9.2 Å (Figure 3b). Different from the structural analogues of **SrPd‐3** and **SrPd‐4**, the propanedioate‐functionalized counterpart could not be obtained in this case, possibly due to the shorter arm of malonic acid. Taking **SrPd‐5** as host, attempts to load a variety of guests (e.g., transition metals, lanthanides, and small organic molecules) into the hydrophilic chamber have been carried out but all failed. Then great efforts were devoted to applying **SrPd‐5** as a giant guest molecule instead. After addition of β‐CD into an aqueous solution containing **SrPd‐5**, another host–guest‐type assembly of [{SrPd_10_O_4_(OH)_4_(H_2_O)(PhAsO_3_)_4_}_2_Pd_4_(C_4_H_4_O_4_)_12_⊂β‐CD]^12−^ (**SrPd‐6**) was harvested. With the help of hydrophobic effects, two of the phenyl groups from **SrPd‐5** penetrate into the macrocycle of β‐CD (Figure 3c). Moreover, every β‐CD holds two phenyl groups from different **SrPd‐5** guests, thus forming a 1D supramolecular chain‐like structure (Figure S8). Formation of such host–guest system was also confirmed by FT‐IR spectroscopy (Figure S9). To assess their solution stability, ESI‐MS and NMR characterizations have been performed afterwards. As shown in Figure 3d, ionic moieties observed at *m*/*z* = 1205.97, 1213.17, 1220.76, 1236.38, 1244.17, and 1251.76 are ascribed to the −5 charged protonated and potassium form of **SrPd‐5** formulated as {H_7_ **SrPd‐5**}^5−^, {H_7_ **SrPd‐5**(H_2_O)_2_}^5−^, {KH_6_ **SrPd‐5**(H_2_O)_2_}^5−^, {K_4_H_3_ **SrPd‐5**}^5−^, {K_5_H_2_ **SrPd‐5**}^5−^, and {K_6_H**SrPd‐5**}^5−^, respectively, suggesting the polyanion has been kept intact in water (Table S1). Moreover, ^1^H and ^13^C NMR spectroscopies have been recorded separately on the ensemble (**SrPd‐6**) and components (**SrPd‐5** and β‐CD) of the host–guest assembly. It turns out that the Pd‐oxo scaffolds have been well preserved for both **SrPd‐5** and **SrPd‐6**, which is in line with the mass data (Figure S10). In particular, ^1^H NMR signals of β‐CD are shifted to a higher field in **SrPd‐6**, supporting no dissociation has been occurred in such host–guest system (Figure 3e).

**Figure 4:**
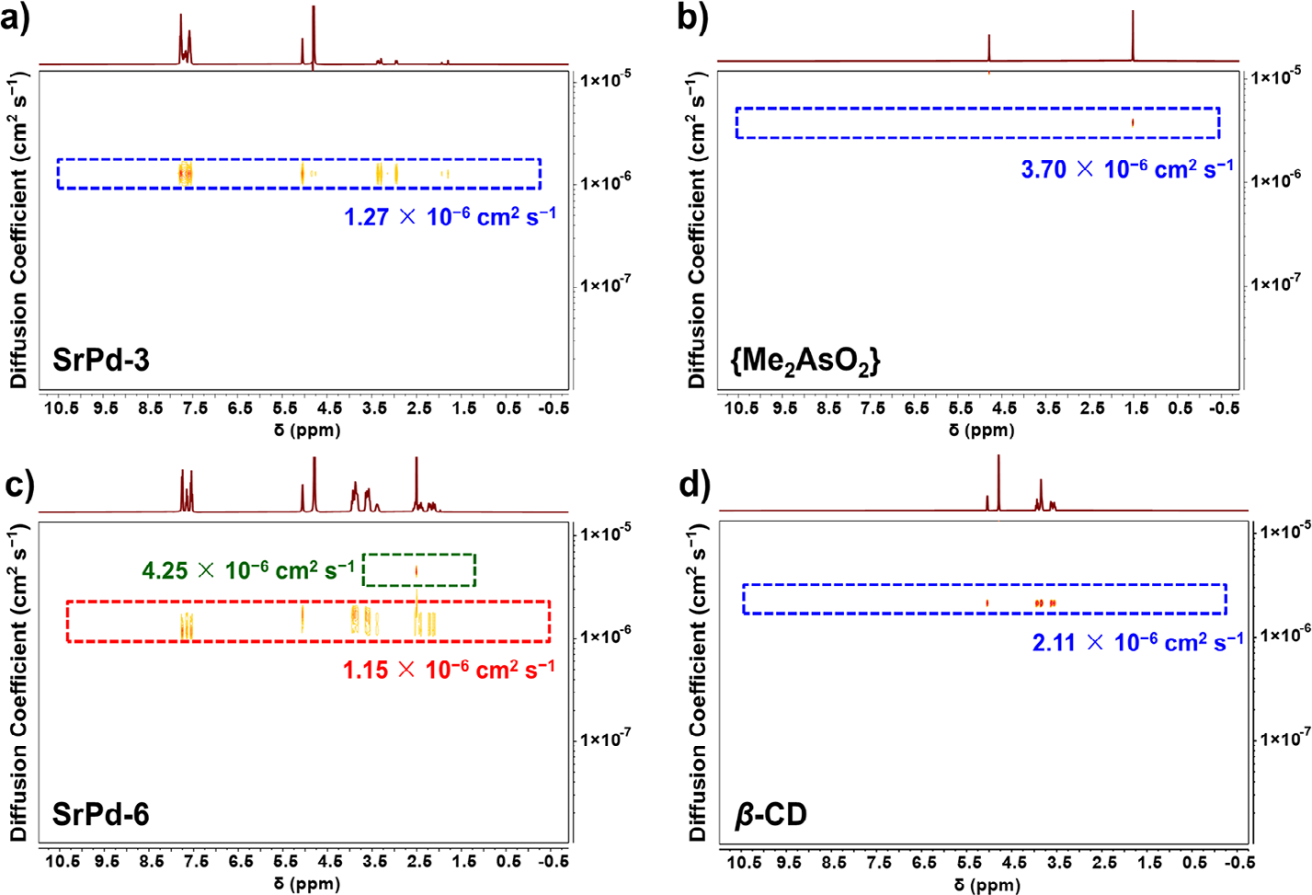
2D ^1^H DOSY NMR spectra of **SrPd‐3** a), sodium dimethylarsinate b), **SrPd‐6** c) and β‐CD d) (60 mM, pH = 7.0, D_2_O, 298 K).

Given the new‐found host–guest patterns in **SrPd‐3/4** and **SrPd‐6**, NMR technique of 2D ^1^H diffusion‐ordered spectroscopy (DOSY) was hired for an in‐depth characterization. For **SrPd‐3**, a single diffusion band with the coefficient value of *D*  =  1.27 × 10^−6^ cm^2^ s^−1^ has been noticed (Figure 4a), which is distinct from that of the independent {Me_2_AsO_2_} (*D*  =  3.70 × 10^−6^ cm^2^ s^−1^, Figure 4b). Similarly, the host and guest components of **SrPd‐4** display almost the same diffusion rate with *D  =* 1.22 × 10^−6^ cm^2^ s^−1^ (Figure S11). The above experimental data strongly signifies the encapsulation of {Me_2_AsO_2_} guest into the bowl‐shaped POP host in solution. With respect to **SrPd‐6**, the signals can be divided into two subgroups (Figure 4c). The diffusion band with *D*  =  1.15 × 10^−6^ cm^2^ s^−1^ corresponds to the entire host–guest assembly (red box). By contrast, the diffusion rate of the free β‐CD is kind of faster (*D*  =  2.11 × 10^−6^ cm^2^ s^−1^, Figure 4d). In addition, the diffusion band with *D*  =  4.25 × 10^−6^ cm^2^ s^−1^ comes from the small amount of co‐crystallized succinate salt (green box). Thus, it can be concluded that the host–guest architecture of **SrPd‐6** is solution stable without dissociation. To deepen the speciation study on the as‐made POPs, their solution stabilities have been further examined by ^1^H NMR under different pH conditions (pH = 5–8) as well as simulated cellular environment (PBS buffer).^[^ ^45^, ^46^ ^]^ As shown in Figures S12–S17, except for the partial dissociation of **SrPd‐3** upon pH fluctuation, the other POPs are all stable under the above conditions. Besides, the long‐term aqueous stability of as‐made POPs has been verified by UV‐vis spectroscopy as well (Figure S18). The constant intensity and position of the absorption bands are indicative of their intact polyanionic frameworks within at least 60 h, which meets the requirement of biological studies.

### Structure‐Dependent Anti‐Tumor Potential

**Figure 5:**
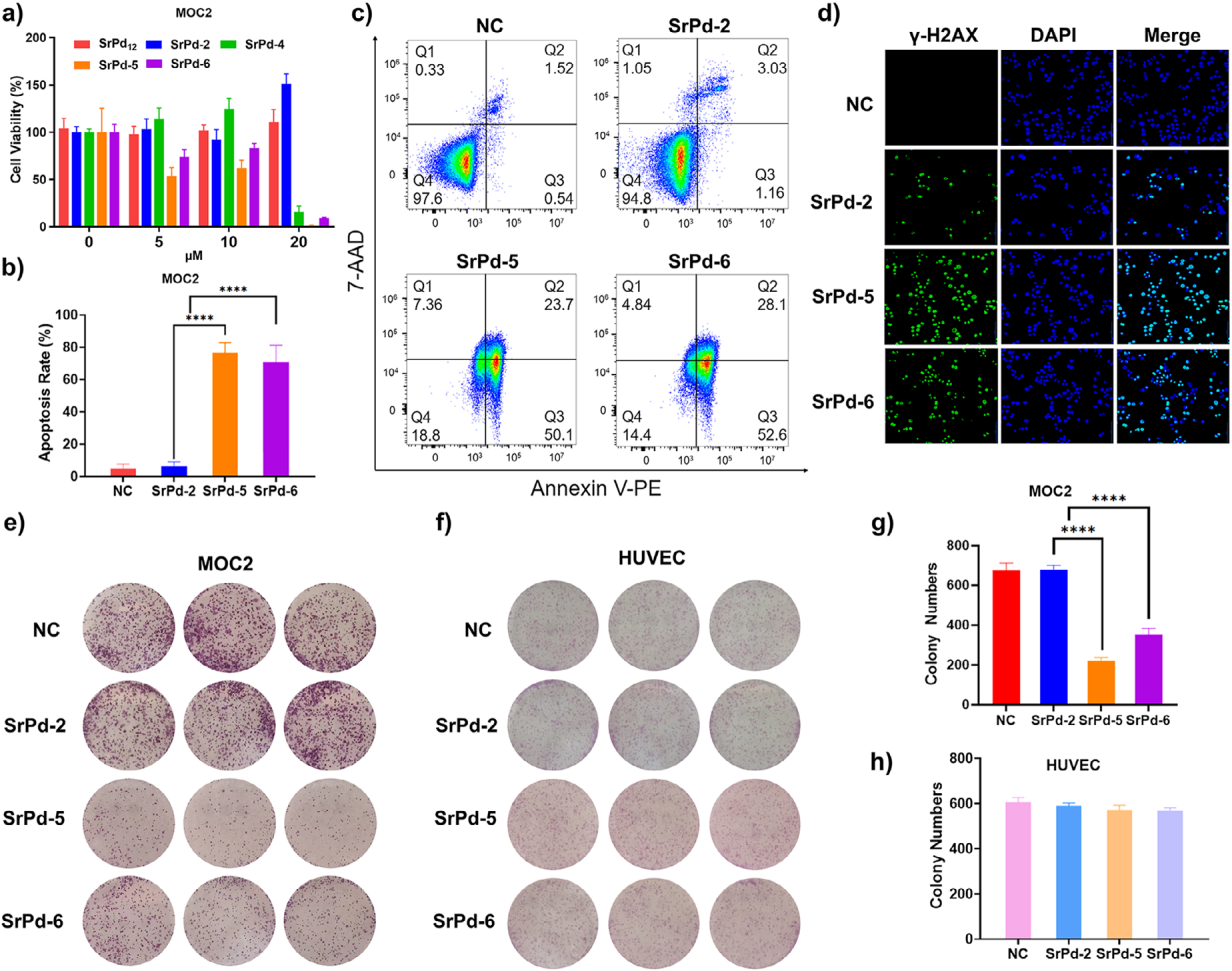
a) CCK‐8 assay on MOC2 cells treated with different POPs for 48 h (*n* = 3, mean ± SD). b) Flow cytometric analysis of the MOC2 cell apoptosis treated with **SrPd‐2** and **SrPd‐5/6** at a concentration of 20 µM (*n* = 3, mean ± SD). ****P< 0.0001. c) Annexin V‐PE/7‐AAD analysis of MOC2 cells treated with **SrPd‐2** and **SrPd‐5/6** at a concentration of 20 µM. d) Immunofluorescence images of γ‐H2AX staining for DNA damage induced by **SrPd‐2** and **SrPd‐5/6**. e)–h) Colony formation assay on MOC2 e), g) and HUVEC f), h) cells treated with **SrPd‐2** and **SrPd‐5/6** (*n* = 3, mean ± SD). ****P < 0.0001. Statistical significance was determined using one‐way ANOVA followed by Tukey's multiple comparisons test.

It has been widely acknowledged that noble metal‐based complexes are of high medicinal values, including but not limited to anti‐tumor, anti‐inflammatory, and anti‐bacteria.^[^ ^47^, ^48^, ^49^ ^]^ Recent studies have unveiled the considerable anti‐tumor activity of **SrPd_12_** against, for example, human neuroblastoma cells.^[^ ^30^ ^]^ While there are still some issues to be solved (e.g., elusive anti‐cancer mechanism) before a clinical trial could be considered. In response to this, cytotoxic effects of the as‐made **SrPd_12_**‐derived POPs with varied configurations have been evaluated. To exclude the interference of organic part, succinate‐functionalized **SrPd‐2/4/5/6** as well as **SrPd_12_** precursor were selected as candidates. Adopting the Cell Counting Kit‐8 (CCK‐8) assay, different inhibitory effects on MOC2 (mouse oral squamous cell carcinoma cell line) cells have been observed. Compared with the primitive **SrPd_12_**, there is no significant difference being found for the open‐shell‐type **SrPd‐2** (Figure 5a). As the concentration increased to 20 µM, a noticeable anti‐tumor effect of the bowl‐shaped **SrPd‐4** has been detected. Among them, the cage‐like **SrPd‐5** and **SrPd‐6** behaved the best performance at various concentrations. In particular, the inhibitory effect of **SrPd‐5** is always better than that of **SrPd‐6**, indicating the packaging of β‐CD might adjust the anti‐tumor activity to a certain extent.

In order to disclose the anti‐tumor mechanism of as‐made POPs in vitro, further experiments have been performed on **SrPd‐2** and **SrPd‐5/6** showcasing the lowest and highest cytotoxicity, respectively. First of all, their abilities to induce apoptosis in tumor cells were assessed using the Annexin V‐PE/7‐AAD apoptosis detection kit. At a concentration of 20 µM, **SrPd‐5** and **SrPd‐6** exhibited significantly enhanced performance as compared to that of **SrPd‐2** (Figure 5b,c). Based on the previous research on noble metal‐based anti‐tumor drugs, the cell death and cytotoxic effect are mostly attributed to DNA damage.^[^ ^50^ ^]^ As a consequence, immunofluorescence staining experiments using γ‐H2AX as DNA damage marker have been conducted. As depicted in Figure 5d, the fluorescence intensity and the number of positive cells were remarkably increased in the cases of **SrPd‐5** and **SrPd‐6**, suggesting their enhanced capacity to induce DNA damage. Additionally, in the cell colony formation tests, **SrPd‐5** and **SrPd‐6** significantly inhibited the proliferation of MOC2 cells as well (Figure 5e,g). On this basis, the cell selectivity of POPs has been preliminarily examined. It has been demonstrated that the chosen POPs exhibited no obvious inhibitory effect on the growth of normal human umbilical vein endothelial cells (HUVEC), as supported by both colony formation (Figure 5f,h) and CCK‐8 assays (Figure S19).

**Figure 6:**
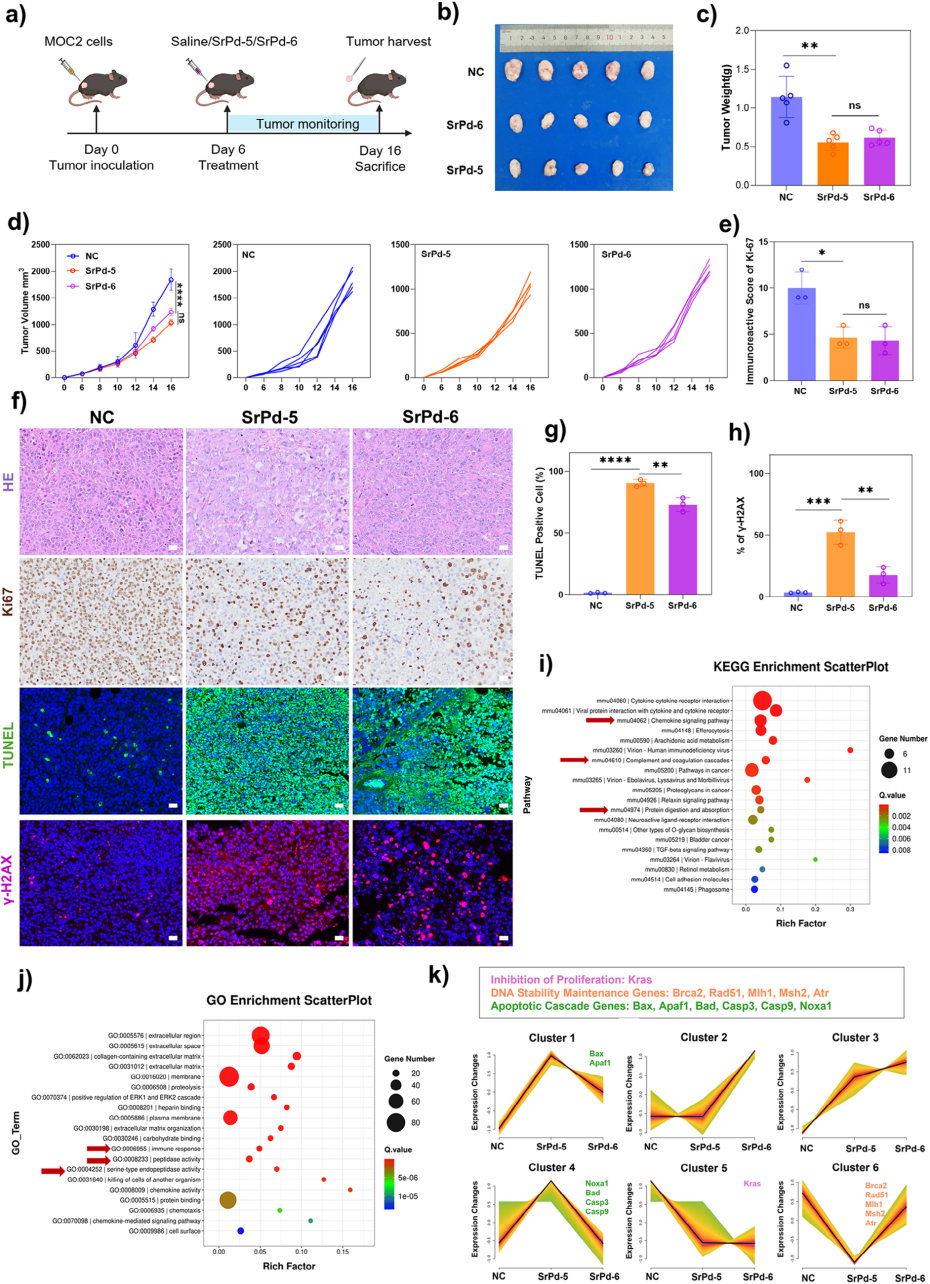
Anti‐tumor efficacy of **SrPd‐5** and **SrPd‐6** in MOC2 tumor‐bearing mice. a) Schematic illustration of the experimental design for the in vivo anti‐tumor study. (Created in BioRender. Liu, Z. (2025) https://BioRender.com/oswd6np) b) Representative photographs of excised tumors from different treatment groups on day 16. c) Tumor weights of different groups at the endpoint (*n* = 5, mean ± SD). **P < 0.01, ns: not significant. d) Tumor growth curves of individual mice and mean tumor volumes in different treatment groups (*n* = 5, mean ± SD). ****P < 0.0001, ns: not significant. e) Quantification of Ki67 immunoreactive scores in tumor sections (*n* = 5, mean ± SD). *P < 0.05, ns: not significant. f) Representative H&E staining, Ki67 immunohistochemistry, TUNEL assay, and γ‐H2AX immunofluorescence images of tumor sections from different groups. Scale bar: 20 µm. g) Quantification of TUNEL‐positive cells in tumor sections (*n* = 5, mean ± SD). ****P < 0.0001, **P < 0.01. h) Quantification of γ‐H2AX‐positive cells in tumor sections (*n* = 5, mean ± SD). ***P < 0.001, **P < 0.01. i) KEGG pathway enrichment analysis of differentially expressed genes between treatment and control groups. j) GO enrichment analysis of differentially expressed genes between treatment and control groups. k) Trend analysis of gene expression patterns showing six distinct clusters of genes involved in proliferation inhibition, DNA damage response, and apoptotic pathways. Statistical analysis was performed using one‐way ANOVA followed by Tukey's multiple comparisons test.

To further validate the anti‐tumor potential of these POPs in vivo, we established a mouse model bearing MOC2 tumor cells. After tumor inoculation, mice were treated with saline (NC group), **SrPd‐5**, and **SrPd‐6**, respectively, on day 6 (Figure 6a).

H&E staining of major organs (heart, liver, spleen, lung, and kidney) showed no obvious pathological changes in the treated groups as compared to the controls, suggesting good biosafety of the as‐made POPs (Figure S20). In comparison with the NC group, both **SrPd‐5** and **SrPd‐6** significantly suppressed the tumor growth, as evidenced by the reduced tumor volume and weight (Figure 6b–d). Immunohistochemical analysis revealed the decreased Ki67 expression in both treatment groups, indicating the reduced tumor cell proliferation (Figure 6f). Quantitative analysis confirmed the significantly lower Ki67‐positive scores in both **SrPd‐5** and **SrPd‐6**‐treated tumors as compared to the controls (Figure 6e). TUNEL staining demonstrated the markedly increased apoptosis in both treatment groups, with **SrPd‐5** inducing the highest level of cell apoptosis (Figure 6f,g). Additionally, γ‐H2AX staining showcased elevated DNA damage in the treated tumors, particularly in the **SrPd‐5** group (Figure 6f,h), corroborating our in vitro mechanistic studies.

Further analysis of transcriptomic profiling disclosed the distinct gene expression patterns in the treated tumors. Obviously, the **SrPd‐5** treatment induced more extensive transcriptional changes (1297 up‐regulated and 174 down‐regulated genes) as compared to the **SrPd‐6** group (636 up‐regulated and 136 down‐regulated genes, Figures S21a and S21b). Hierarchical clustering analysis demonstrated the consistent and treatment‐specific gene expression signatures, with **SrPd‐5** displaying more pronounced changes that align with its outstanding anti‐tumor efficacy (Figures S21c–S21e). Comparative pathway analysis further revealed several shared molecular signatures between **SrPd‐5** and **SrPd‐6** treatments as compared to NC. Both treatments affected extracellular matrix components, calcium signaling pathway, and membrane‐associated processes, indicating their potential influence on cell–cell interactions and signal transduction. These common molecular signatures provide mechanistic insights into their shared anti‐tumor properties (Figures S21f and S21 g). In addition, KEGG and GO enrichment analyses highlighted the distinct gene expression patterns between **SrPd‐5** and **SrPd‐6** treatments as compared to NC. KEGG analysis highlighted the cytokine–cytokine receptor interaction and chemokine signaling pathways as key differences between the two POPs (Figure 6i). GO enrichment indicated that **SrPd‐5** uniquely regulated the extracellular matrix organization and ERK1/2 cascade pathways as compared to **SrPd‐6** (Figure 6j). Notably, fuzzy clustering analysis identified six distinct gene expression patterns across the treatments (Figure 6k). Clusters 1 and 4 showed peaked expression in **SrPd‐5**‐treated samples, enriched with apoptotic cascade genes (Bax, Apaf1, Bad, Casp3). Importantly, Cluster 6 exhibited a distinctive V‐shaped expression pattern across the treatment groups, characterized by the significant downregulation in **SrPd‐5**‐treated samples followed by a marked recovery in the **SrPd‐6**‐treated samples as compared to NC. This cluster predominantly contains the DNA stability maintenance genes, including Brca2, Rad51, Mlh1, Msh2, and Atr. The pronounced differential regulation of these DNA repair‐associated genes suggests the distinct effects of **SrPd‐5** and **SrPd‐6** on the genomic stability mechanisms. The sharp suppression of these genes by **SrPd‐5** may indicate its potential to compromise DNA repair capacity in the tumor cells, while their upregulation by **SrPd‐6** suggests a possible compensatory DNA repair response. These in vivo results support our findings that the cage‐like POPs, in particular **SrPd‐5**, exhibit potent and selective anti‐tumor activity through the induction of DNA damage and subsequent cell death, while maintaining a favorable safety profile.

Interestingly, the anti‐tumor activity of as‐made POPs has been remarkably promoted along with the structural evolution, especially for **SrPd‐5** and **SrPd‐6**. According to the experimental results, it is plausible that the size of POPs might affect their anti‐tumor performances. From open‐shell via bowl to cage‐like POPs, the nuclearity of Pd^II^ addenda has been increased from 12 to 24 along with an obvious volume expansion (Figure S22). Similar size effect has also been noted for the cluster‐based anti‐cancer pharmaceuticals.^[^ ^51^, ^52^ ^]^ As compared to **SrPd‐5**, the Pd‐oxo cluster in **SrPd‐6** has been partially wrapped by β‐CDs, which may enhance the steric hindrance and impede the interactions between POP and DNA, followed by the difference in bioactivity. According to the reported works, the interactions between POMs and DNA have been observed before.^[^ ^53^, ^54^ ^]^ The bindings of POMs toward DNA might depend on a non‐covalent groove or outside stacking mode.^[^ ^55^ ^]^ As referenced by the above, **SrPd‐5** may affect the genomic stability via a non‐intercalated mode, in view of the negatively charged attribute of POP and DNA. In addition, the acidic tumor microenvironment potentially enhances the attraction of the polyanionic POPs, thereby offering advantages over traditional receptor‐ligand targeting strategies (Figure S23).^[^ ^56^ ^]^ This also explains the selective targeting of POPs on tumor cells rather than normal tissues. Besides, except for the DNA damage, the other possible anti‐tumor pathways of POPs have also been considered. Based on the extensive literature research, the proposed modes of action and biological targets of anti‐tumor POMs are many and varied. ^[^ ^35^, ^57^, ^58^ ^]^ The most common anti‐tumor routes involve inhibition of ATP generation and increase of ROS‐level. In contrast to the W^V/VI^ and Mo^V/VI^ addenda in polyoxo‐tungstates and ‐molybdates that possess reversible redox activity, the Pd^II^ addenda in POPs are poor at electron transfer, which has been demonstrated by the electrochemical characterizations.^[^ ^25^ ^]^ Therefore, the as‐made POPs are unlikely to interfere the ATP generation by influencing the intracellular electron transfer chain. Secondly, as reported in our latest research, some of POPs could promote the ROS production and induce the apoptosis of tumor cells of different types.^[^ ^34^ ^]^ It has been revealed that the heterogroups of POPs play a crucial role in determining the ROS generation. For example, more ROS have been detected in the system of {AsO_4_}‐capped POPs rather than that in the {PO_4_}‐capped counterparts. Considering the accumulation of H_2_O_2_ in the tumor microenvironment, the production capacity of hydroxyl radical has been examined by TMB (3,3′,5,5′‐tetramethylbenzidine) chromogenic method. As illustrated in Figure S24, the concentration of hydroxyl radical is very low under the simulated cellular environment. This indicates that the increasement of ROS‐level might not be the key for anti‐tumor process. In contrast, both in vitro and in vivo studies have demonstrated unambiguously the increase of γ‐H2AX marker and inhibition of repair gene, indicating DNA is recognized as the main target of POPs in this case. It is thus believed that the imbalance of damage and repair of DNA might be responsible for interpreting the anti‐tumor activity of as‐made POPs.

## Conclusion

The post‐modification of **SrPd_12_** as MBB has succeeded in switching on a highly ordered structural evolution of POPs. By means of ligand replacement and host–guest recognition, unprecedented bowl‐ and cage‐like archetypes as well as their supramolecular aggregates have been unlocked. Of these, covalent (coordination bond) and non‐covalent (hydrogen bond and hydrophobic effect) forces have been tamed for building such sophisticated hierarchical assemblies, which is strikingly similar to the construction management of biomacromolecules intended by nature. Apart from a series of findings with structural interests, including the anion@anion‐type host–guest system and penta‐vacant building unit, the structure‐dependent anti‐tumor activities have been studied in detail. Both in vitro and in vivo anti‐tumor studies have revealed that the significant inhibition against MOC2 cells can be mainly attributed to the DNA damage. In contrast to the bioactive tungsten/molybdenum‐based POMs that aim at ATP inhibition or ROS generation, the dual function of **SrPd‐5**, namely induction of damage and inhibition of repair, makes it a promising anti‐tumor candidate that takes DNA as target, diversifying the anti‐tumor pathways of POM‐based pharmaceuticals of the next generation.

## Conflict of Interests

The authors declare no conflict of interest.

## Supporting information



Supplementary Information

Supplementary Information

## Data Availability

The data that support the findings of this study are available in the supplementary material of this article.
