# Supplementary material for: Biomimetic Hierarchical Construction of Anti‐Tumor Polyoxopalladates for Cancer Therapy
Source: Angew Chem Int Ed Engl. 2025 Apr 14;64(23):e202505564. doi: 10.1002/anie.202505564 (PMC12124443; doi:10.1002/anie.202505564)
Supplement: Supplementary file 1 — Supplementary Information [file ANIE-64-e202505564-s002.pdf]

Supporting Information

Biomimetic Hierarchical Construction of Anti-Tumor Polyoxopalladates for Cancer Therapy

Yue Zhao,[a]# Zheran Liu,[b]# Zijian Qin,[b] Qinlong Wen,[b] Jing Du,[d] Xiang-Yu Ren,[a] Chao-Qin Chen,[a] Xingchen Peng,*[b] Ulrich Kortz,*[c] and Peng Yang*[a]

a College of Chemistry and Chemical Engineering
Hunan University, 410082 Changsha, P. R. China
E-mail: pengyang216@hnu.edu.cn

b Department of Biotherapy, Cancer Center
West China Hospital, Sichuan University, 610041 Chengdu, P. R. China
E-mail: pxx2014@scu.edu.cn

c School of Science
Constructor University, Campus Ring 1, 28759 Bremen, Germany
E-mail: ukortz@constructor.university

d Testing and Analysis Center
Hebei Normal University, 050024 Shijiazhuang, P. R. China

Table of Contents (28 pages)

1. Experimental Details S2
2. Synthesis of Compounds S5
3. Characterizations on Compounds S7
4. References S28

1. Experimental Details

1.1 Materials:

The precursor of $Na_4[SrPd_{12}O_6(OH)_3(PhAsO_3)_6(OAc)_3]\cdot 2NaOAc\cdot 32H_2O$ (**Na-SrPd$_{12}$**)[S1] was synthesized according to the published procedure and the identity of the product was confirmed by FT-IR spectroscopy. Genomic DNA from human epithelial cells was isolated using the Genomic DNA Mini Preparation Kit with Spin Column (D0063 Magne, Beyotime), according to the manufacturer's protocol. All other chemicals and reagents were purchased from commercial suppliers and used without further purification.

1.2 Physical Measurements:

FT-IR. The Fourier transform infrared (FT-IR) spectra were recorded on KBr disk using a Shimadzu IR Spirit-T spectrometer between 400 and 4000 cm^{-1}.

Elemental Analyses. CHN microanalyses were performed on a Perkin-Elmer 240C elemental analyzer, and ICP-OES analyses were performed on a Perkin-Elmer Optima 8300 optical emission spectrometer.

TGA. Thermogravimetric analyses (TGA) were carried out on a TA Instruments SDT Q600 thermobalance with a 100 mL min^{-1} flow of nitrogen; the temperature was ramped from 25 to 800 °C at a rate of 5 °C min^{-1}.

NMR. The ^1H and ^{13}C nuclear magnetic resonance (NMR) spectra were recorded on a Bruker Avance III 400 MHz instrument at room temperature, using 5-mm tubes for ^1H and ^{13}C with respective resonance frequencies of 399.78 MHz (^1H) and 100.71 MHz (^{13}C).

ESI-MS. The electrospray-ionization mass spectrometry (ESI-MS) measurements were made in the negative ion mode on an Agilent 6520 Q-TOF LC/MS mass spectrometer coupled to an Agilent 1200 LC system, and all the MS data were processed by the MassHunter Workstation software. Sample solutions were ca. 10^{-5} M in water and were transferred to the electrospray source by direct injection.

UV-vis Absorption. The ultraviolet-visible (UV-vis) absorption spectra were measured at room temperature using a Shimadzu UV-1900i spectrophotometer.

X-ray Crystallography. Single crystals of the six compounds were mounted in a Hampton cryoloop with light oil to prevent efflorescence. Data collections were performed at 150 K on a Bruker D8 Quest single-crystal diffractometer equipped with Mo *K*α radiation (λ = 0.71073 Å). All structures were solved with the ShelXT structure solution program using Intrinsic Phasing[S2] and refined with the ShelXL refinement package using Least Squares minimization[S3] operated in the OLEX2 interface.[S4] All non-hydrogen atoms were refined anisotropically. It was not possible to locate all counter cations by X-ray diffraction, probably due to crystallographic disorder, which is a common problem in POM crystallography. Thus, the SQUEEZE program[S5] or the Olex2 solvent mask function were further used to remove the contributions of weak reflections from the whole data. The newly generated hkl data were further used to refine the final crystal data. Therefore, the exact number of counter cations and solvent molecules was determined by elemental analysis and thermogravimetric diagrams. The resulting formula units were further used throughout the paper. In the Supporting Information, the crystal data and structure refinement for the six compounds is summarized in Table S3.

1.3 Anti-tumor Evaluation:

Cell Culture

MOC2 and HUVEC cell lines were utilized for this study. These cells were cultured in Dulbecco's Modified Eagle Medium (DMEM) supplemented with 10% fetal bovine serum (FBS) and maintained in a humidified incubator at 37 °C with 5% CO_2.

Cytotoxicity Assay

Cells were seeded in 96-well plates at 5×10^3 cells/well and incubated at 37°C in a 5% CO_2 atmosphere for 24 hours and then treated with **SrPd-2**, **SrPd-5** and **SrPd-6**. After 48 hours, CCK-8 solution (Oriscience Cell Counting Kit-8, No. CB101) was added to the culture medium at a 1:10 ratio and incubated at 37 °C for 2 hours before measuring the absorbance at 450 nm.

Flow Cytometric Analysis of Apoptosis

Apoptosis was assessed using the Annexin V-PE/7-AAD Apoptosis Detection Kit (Annexin V-PE/7-AAD Apoptosis Detection Kit, Vazyme, No. A213-02). MOC2 cells were seeded in 6-well plates at 2×10^5 cells/well and incubated at 37 °C in a 5% CO_2 atmosphere for 24 hours. Post-treatment with **SrPd-2**, **SrPd-5** and **SrPd-6** (20 μM each), cells were harvested 24 hours later, washed twice with precooled PBS, and incubated in the dark with Annexin V-PE and 7-AAD for 10 minutes before flow cytometric analysis.

γ-H2AX Immunofluorescence Staining

DNA damage was detected using γ-H2AX Immunofluorescence Staining (DNA Damage Assay Kit by γ-H2AX Immunofluorescence, Beyotime, No. C2035S). MOC2 cells were seeded in 96-well plates at 5×10^3 cells/well and incubated at 37 °C in a 5% CO_2 atmosphere for 24 hours and then treated with **SrPd-2**, **SrPd-5** and **SrPd-6** (10 μM each). After 24 hours, cells were washed with PBS, fixed for 30 minutes, incubated overnight at 4 °C with γ-H2AX rabbit monoclonal antibody, followed by incubation with Alexa Fluor 488-conjugated anti-rabbit secondary antibody for 1 hour at room temperature in the dark. After two washes, cells were stained with DAPI for 10 minutes at room temperature and imaged using a confocal microscope.

Colony Formation Assay

Cells were seeded in 6-well plates at a density of 1×10^3 cells/well and incubated at 37 °C in a 5% CO_2 atmosphere for 24 hours. The cells were then treated with **SrPd-2**, **SrPd-5** and **SrPd-6** (20 μM each). Post-irradiation, cells were washed with PBS after 24 hours and further incubated with fresh DMEM containing 10% FBS for 6-7 days until visible colonies formed. The colonies were then fixed with 4% paraformaldehyde for 30 minutes and stained with 1% crystal violet.

Animals

Female BALB/c mice (6-8 weeks old) were used to establish a MOC2 tumor xenograft model. MOC2 cells (3×10^5) were subcutaneously injected into the right flank of mice. When tumor volumes reached approximately 50-80 mm^3 (day 6 post-inoculation), mice were randomly divided into three groups (n=5 per group): control group (saline), **SrPd-5** group, and **SrPd-6** group. The POPs were administered intratumorally at a dose of 0.4 mg per mouse. Tumor dimensions were measured every 3 days using digital calipers, and volumes were calculated using the formula: $V = (\text{length} \times \text{width}^2)/2$. Body weights were monitored throughout the study period to assess potential toxicity. Mice were humanely sacrificed when the largest tumor in the control group reached 2000 mm^3. Tumors were harvested, weighed, and processed for subsequent histological and molecular analyses.

Ethical Statement

All animal procedures were performed in accordance with protocols approved by the institutional animal care and use committee. All procedures were conducted in accordance with institutional guidelines and approved by the Animal Ethics Committee, West China Hospital of Sichuan University (No.20241017001).

Histological and Immunological Analyses

Harvested tumor tissues and major organs (heart, liver, spleen, lung, and kidney) were fixed in 4% formalin for 48 hours, embedded in paraffin, and sectioned into 4 μm slices. For H&E staining, deparaffinized sections were stained with hematoxylin and eosin following standard protocols to assess tissue morphology and potential toxicity. For immunohistochemistry (IHC), tissue sections were incubated with primary antibodies against Ki67 (1:1000; GB111499, Servicebio) or γ-H2AX (1:100; GB111841, Servicebio), followed by HRP-conjugated Goat Anti-Rabbit IgG (1:200, GB23303, Servicebio). The immunoreactivity was visualized using diaminobenzidine (DAB, G1212, Servicebio). Quantitative analysis was performed by calculating immunoreactive scores, determined by multiplying the percentage score (PS: 0-4, based on percentage of positive cells) and intensity score (IS: 0-3, based on staining intensity).

Apoptosis and DNA Damage Analyses

Apoptosis was evaluated using the TUNEL assay according to the manufacturer's protocol. For immunofluorescence analysis, sections were stained with γ-H2AX antibody (1:100; GB111841, Servicebio) and visualized using Cy3-conjugated Donkey Anti-Goat IgG (1:300; GB21303, Servicebio).

RNA Sequencing and Bioinformatics Analyses

Total RNA was extracted from tumor tissues using TRIzol Reagent (Thermofisher, 15596018) according to manufacturer's instructions. RNA sequencing was performed by LC-Biotechnology CO., Ltd. (Hangzhou, China). Transcriptomic data analysis was conducted using R software. Differential gene expression analysis was performed using DESeq2 package, with criteria of $|\log2FoldChange| > 1$ and adjusted $P\text{-value} < 0.05$. Pathway enrichment analyses, including KEGG and GO analyses, were performed using the clusterProfiler package to identify significantly altered biological processes and molecular pathways. Fuzzy clustering analysis was conducted using the Mfuzz package to identify distinct gene expression patterns across treatment groups, with special focus on DNA repair and apoptosis-related genes.

2. Synthesis of Compounds

Synthesis of $Na_7[SrPd_{12}O_6(OH)_3(PhAsO_3)_6(C_3H_2O_4)_3]\cdot 27H_2O$ (Na-SrPd-1)

Na-SrPd12 (0.023 g, 0.006 mmol) was dissolved in 2 mL of 0.5 M sodium propanedioate solution (pH 7.0). While stirring, the solution was heated to 50 °C for 90 min. Then the solution was allowed to cool to room temperature, filtered, and the filtrate was left for crystallization at room temperature in an open vial. Dark red, rodlike crystals were obtained after one week, and were collected by filtration followed by air-drying. Yield: 0.017 g (71% based on **Na-SrPd12**). Elemental analysis (%): Calcd: C 14.75, Na 4.39, As 12.26, Sr 2.39, Pd 34.84. Found: C 14.33, Na 4.68, As 11.93, Sr 2.69, Pd 35.22. FT-IR (2% KBr pellet, ν/cm^{-1}): 3389 (br), 1542 (s), 1438 (s), 1348 (s), 1261 (w), 1184 (w), 1093 (s), 813 (s), 748 (m), 696 (m), 534 (s).

Synthesis of $K_2Na_3[SrPd_{12}O_6(OH)_3(PhAsO_3)_6(C_4H_4O_4)(OAc)_2]\cdot 42H_2O$ (KNa-SrPd-2)

This compound was prepared by exactly the same procedure as **Na-SrPd-1**, but 0.5 M potassium succinate solution (pH 7.0) was used instead of 0.5 M sodium propanedioate solution (pH 7.0). The solution was heated to 50 °C under stirring for 90 minutes. Then the solution was allowed to cool to room temperature, filtered, and the filtrate was left for crystallization at room temperature in an open vial. Dark red, rodlike crystals were obtained after one week, and were collected by filtration followed by air-drying. Yield: 0.017 g (74% based on **Na-SrPd12**). Elemental analysis (%): Calcd: C 13.73, Na 1.79, K 2.03, As 11.68, Sr 2.28, Pd 33.17. Found: C 14.15, Na 1.53, K 1.98, As 11.42, Sr 1.98, Pd 33.84. FT-IR (2% KBr pellet, ν/cm^{-1}): 3399 (br), 1637 (m), 1545 (s), 1399 (s), 1303 (m), 1098 (m), 825 (s), 688 (w), 523 (s).

Synthesis of $K_7Na_2[(C_2H_6AsO_2)\subset\{SrPd_{12}O_6(OH)_3(PhAsO_3)_6\}_2(C_3H_2O_4)_3]\cdot 53H_2O$ (KNa-SrPd-3)

Na-SrPd12 (0.023 g, 0.006 mmol) and sodium cacodylate (0.022 g, 0.1 mmol) were dissolved in 2 mL of 0.5 M potassium propanedioate solution (pH 7.0). The solution was heated to 50 °C under stirring for 30 minutes and then pH of the solution was adjusted to 6.7 – 7.3 by addition of 1 M KOH solution. The resulting solution was heated at 50 °C for another 90 minutes. Then the solution was allowed to cool to room temperature, filtered, and the filtrate was left for crystallization at room temperature in an open vial. Dark red, plate-shaped crystals were obtained after one week, and were collected by filtration followed by air-drying. Yield: 0.006 g (28% based on **Na-SrPd12**). Elemental analysis (%): Calcd: C 13.96, K 3.83, Na 0.64, As 13.64, Sr 2.45, Pd 35.77. Found: C 14.35, K 3.45, Na 0.73, As 13.32, Sr 2.39, Pd 36.27. FT-IR (2% KBr pellet, ν/cm^{-1}): 3400 (br), 1636 (m), 1572 (s), 1438 (s), 1350 (s), 1095 (s), 812 (s), 538 (s).

Synthesis of $K_7Na_3[(C_2H_6AsO_2)\subset\{SrPd_{12}O_6(OH)_3(PhAsO_3)_6\}_2(C_4H_4O_4)_3]\cdot 0.5C_4H_4O_4\cdot 55H_2O$ (KNa-SrPd-4)

Na-SrPd12 (0.023 g, 0.006 mmol) and sodium cacodylate (0.022 g, 0.1 mmol) dissolved in 2 mL of 0.5 M potassium succinate solution (pH 7.0). The solution was heated to 50 °C under stirring for 30 minutes and then pH of the solution was adjusted to 7.0 – 9.0 by addition of 1 M KOH solution. The resulting solution was heated at 50 °C for another 90 minutes. Then the solution was allowed to cool to room temperature, filtered, and the filtrate was left for crystallization at room temperature in an open vial. Dark red, plate-shaped crystals were obtained after one week, and were collected by filtration followed by air-drying. Yield: 0.009 g (41% based on **Na-SrPd12**). Elemental analysis (%):

Calcd: C 14.48, K 3.75, Na 0.94, As 13.34, Sr 2.40, Pd 34.99. Found: C 14.15, K 4.03, Na 0.65, As 13.66, Sr 2.21, Pd 35.31. FT-IR (2% KBr pellet, ν/cm^{-1}): 34011 (br), 2169 (w), 1633 (m), 1547 (s), 1427 (s), 1303 (s), 1183 (m), 1095 (s), 809 (w), 536 (s).

Synthesis of $K_{13}[\{SrPd_{10}O_4(OH)_4(H_2O)(PhAsO_3)_4\}_2Pd_4(C_4H_4O_4)_{12}]\cdot0.5C_4H_4O_4\cdot59H_2O$ (K-SrPd-5)

Na-SrPd$_{12}$ (0.023 g, 0.006 mmol) and $Pd(OAc)_2$ (0.011 g, 0.050 mmol) dissolved in 2 mL of 0.5 M potassium succinate solution (pH 7.0). The solution was heated to 50 °C under stirring for 30 minutes and then pH of the solution was adjusted to 5.4 – 5.7 by addition of 1 M HNO_3 solution. The resulting solution was heated at 50 °C for another 90 minutes. Then the solution was allowed to cool to room temperature, filtered, and the filtrate was left for crystallization at room temperature in an open vial. Dark red, block crystals were obtained after one week, and were collected by filtration followed by air-drying. Yield: 0.005 g (22% based on **Na-SrPd$_{12}$**). Elemental analysis (%): Calcd: C 15.38, K 6.64, As 7.83, Sr 2.29, Pd 33.38. Found: C 14.96, K 6.33, As 7.65, Sr 2.03, Pd 33.65. FT-IR (2% KBr pellet, ν/cm^{-1}): 3401 (br), 1560 (s), 1385 (m), 1293 (m), 1172 (m), 1095 (m), 997 (m), 812 (s), 536 (s).

Synthesis of $K_{13}[\{SrPd_{10}O_4(OH)_4(H_2O)(PhAsO_3)_4\}_2Pd_4(C_4H_4O_4)_{12}\subset\beta\text{-CD}]\cdot0.5C_4H_4O_4\cdot88H_2O$ (K-SrPd-6)

K-SrPd-5 (0.046 g, 0.006 mmol) and *β*-CD (0.050 g, 0.044 mmol) dissolved in 2 mL of 0.5 M potassium succinate solution (pH 7.0). The solution was heated to 50 °C under stirring for 30 minutes and then pH of the solution was adjusted to 4.8 – 5.3 by addition of 1 M HNO_3 solution. The resulting solution was heated at 50 °C for another 90 minutes. Then the solution was allowed to cool to room temperature, filtered, and the filtrate was left for crystallization at room temperature in an open vial. Dark red, plate-shaped crystals were obtained after one week, and were collected by filtration followed by air-drying. Yield: 0.005 g (18% based on **K-SrPd-5**). Elemental analysis (%): Calcd: C 18.06, K 5.46, As 6.44, Sr 1.88, Pd 27.44. Found: C 18.25, K 4.99, As 6.23, Sr 2.07, Pd 28.16. FT-IR (2% KBr pellet, ν/cm^{-1}): 3413 (br), 1562 (s), 1380 (s), 1293 (w), 1160 (m), 1027 (m), 806 (s), 530 (s).

3. Characterizations on Compounds

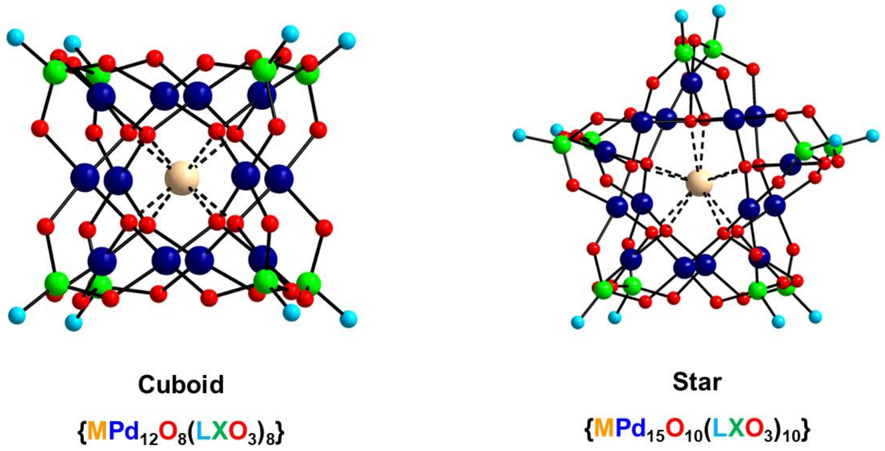

Fig. S1. Structural representation of the cuboid $[MPd_{12}O_8(LXO_3)_8]^{n-}$ and star-like $[MPd_{15}O_{10}(LXO_3)_{10}]^{n-}$ structural models of POPs.

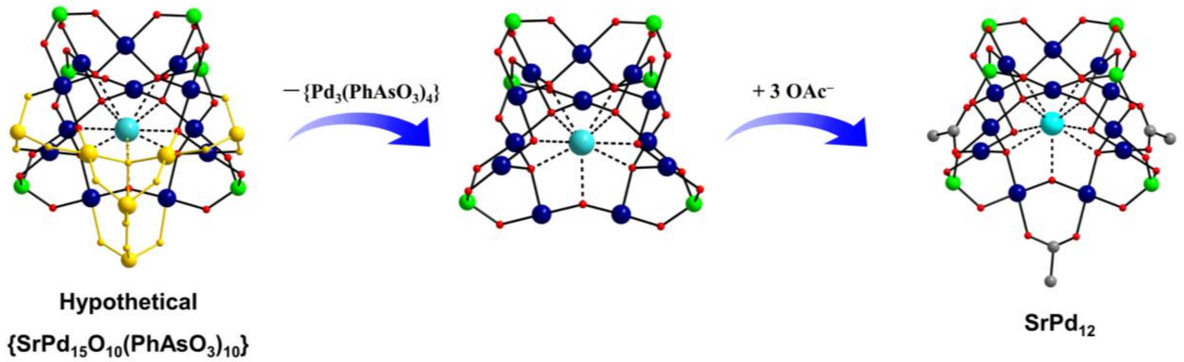

Fig. S2. Structural evolution from the hypothetical $\{SrPd_{15}O_{10}(PhAsO_3)_{10}\}$ to **$SrPd_{12}$**. Phenyl groups are omitted for clarity.

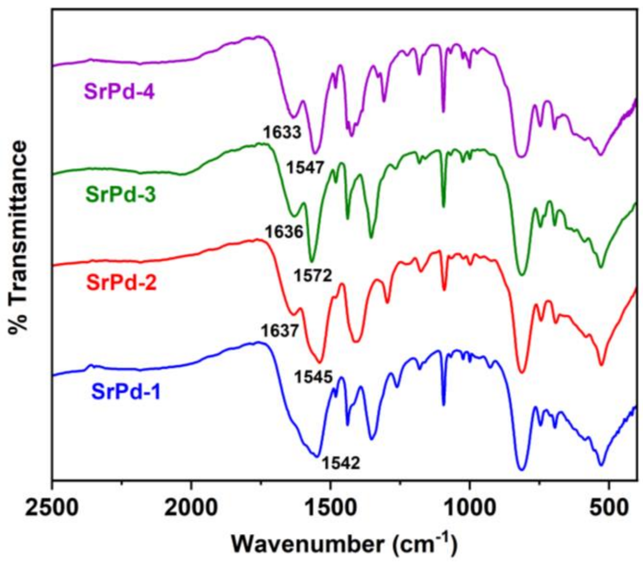

Fig. S3. FT-IR spectra of **SrPd-1/2/3/4**.

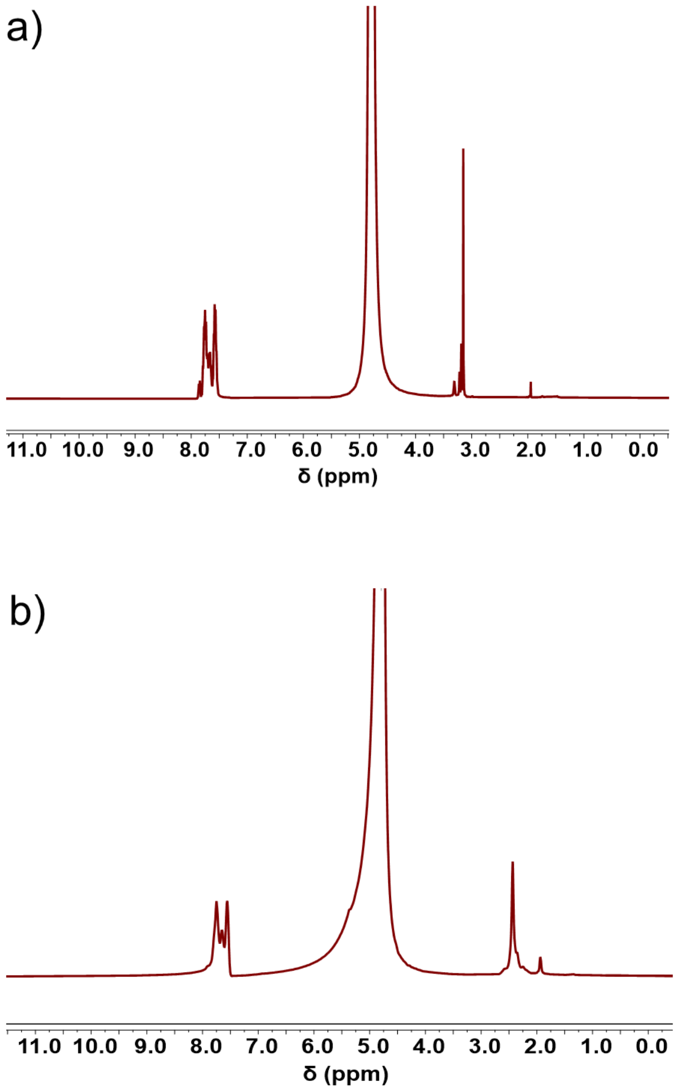

Fig. S4. ^{1}H NMR spectra of **SrPd-1** (a) and **SrPd-2** (b) (20 mM, pH = 7.0, D_2O, 298K).

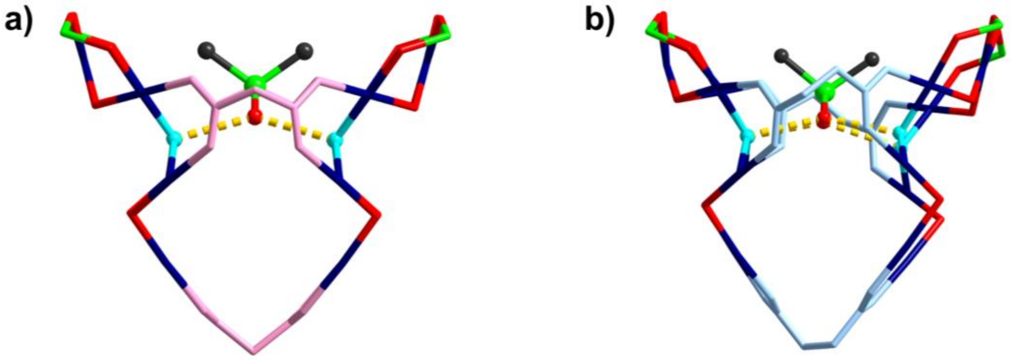

Fig. S5. Position of {Me_2AsO_2} guest inside **SrPd-3** (a) and **SrPd-4** (b).

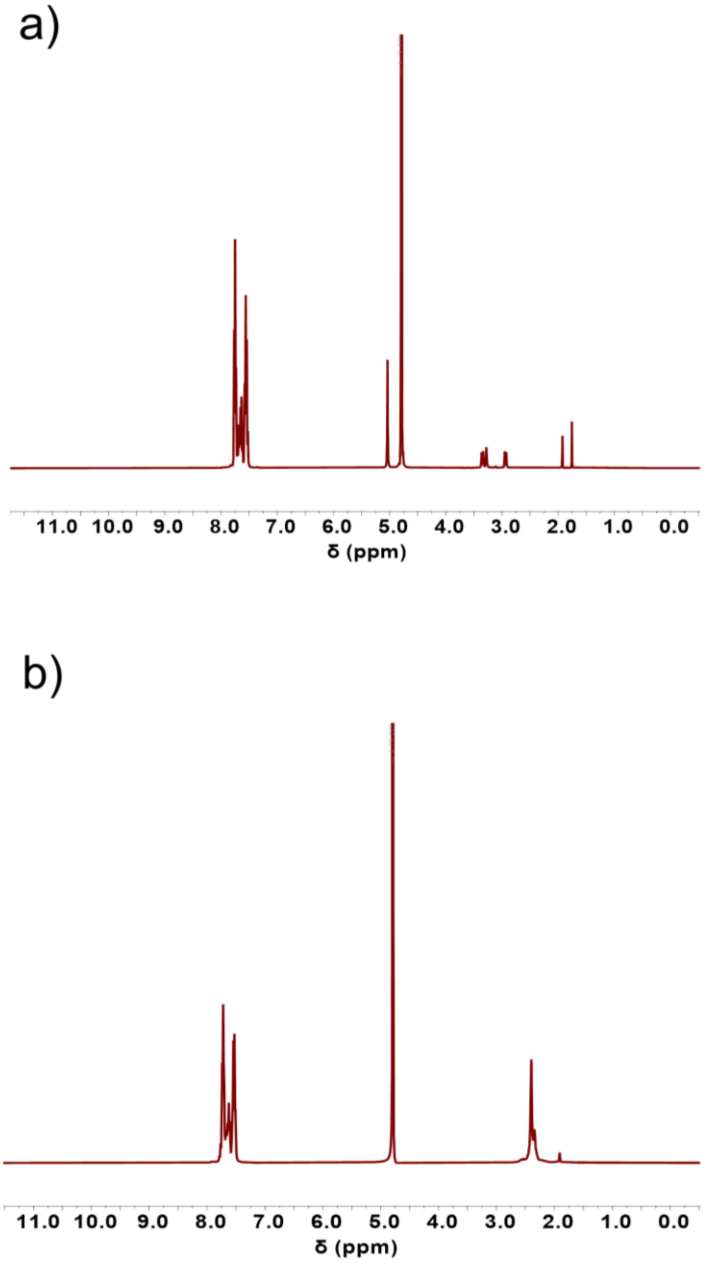

Fig. S6. ^{1}H NMR spectra of **SrPd-3** (a) and **SrPd-4** (b) (20 mM, pH = 7.0, D_2O, 298K).

a)

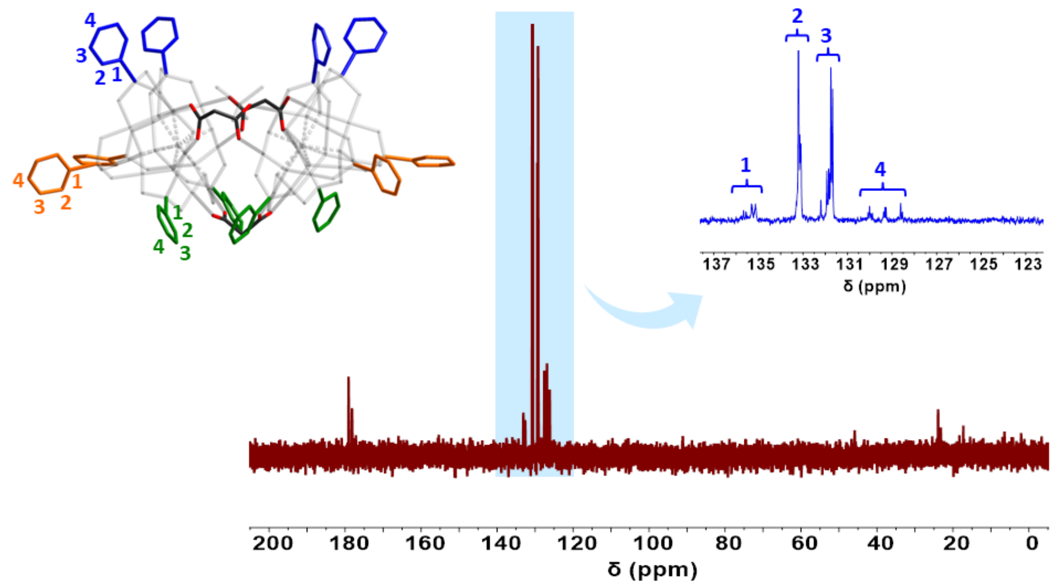

b)

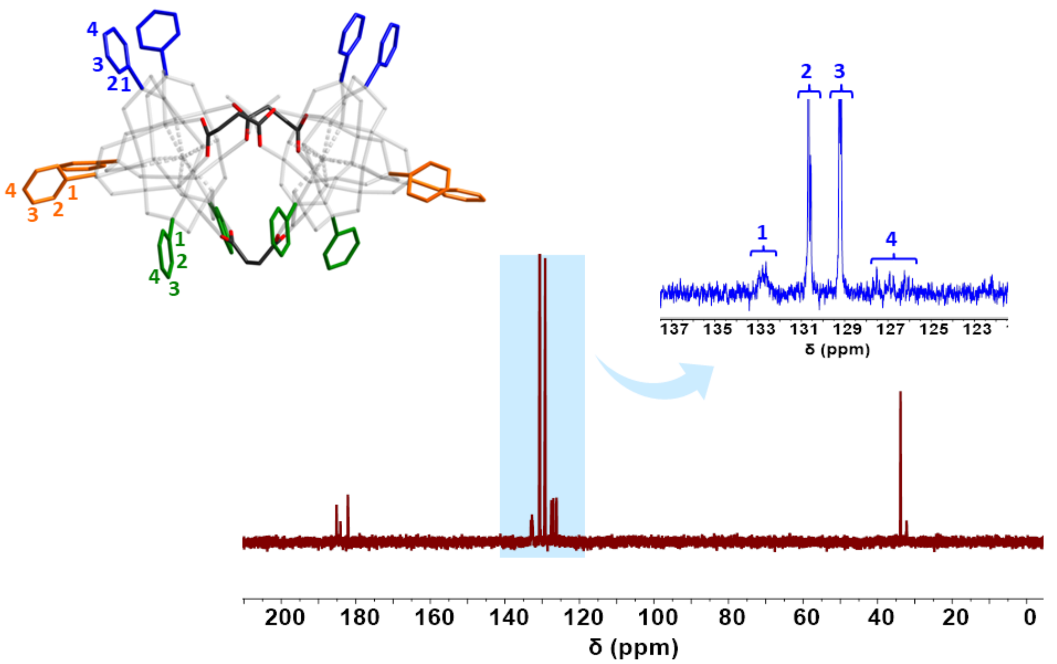

Fig. S7. ^{13}C NMR spectra of **SrPd-3** (a) and **SrPd-4** (b) (100 mM, pH = 7.0, D_2O, 298K).

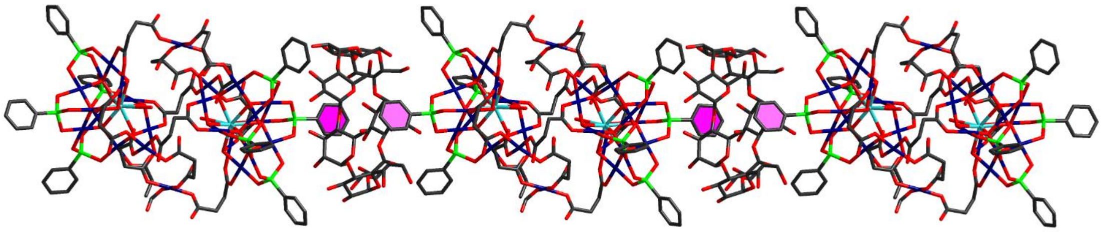

Fig. S8. 1D supramolecular chain-like structure of **SrPd-6**.

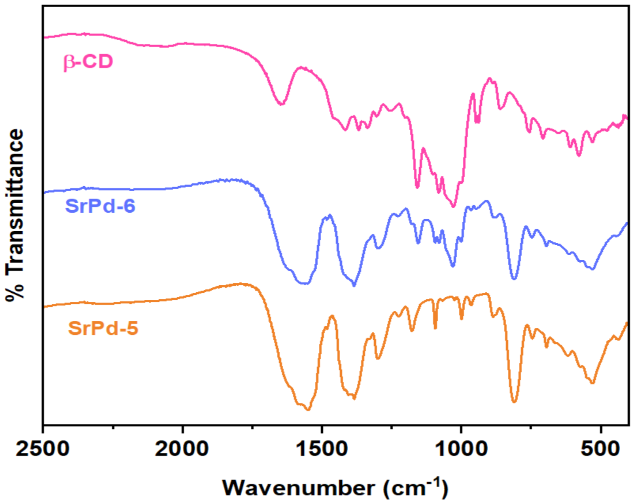

Fig. S9. FT-IR spectra of **SrPd-5**, **SrPd-6**, and *β*-CD.

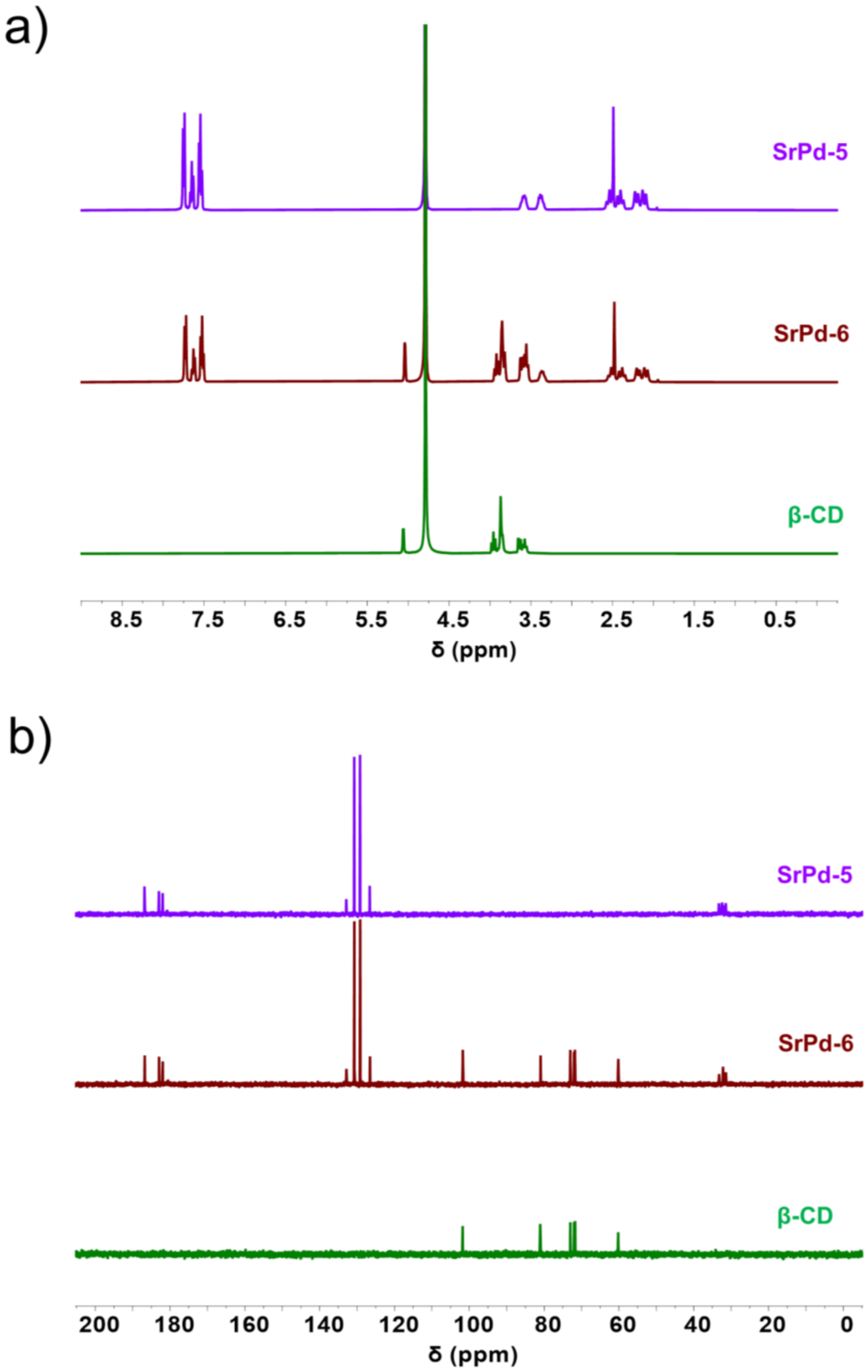

Fig. S10. ^{1}H (a) and ^{13}C (b) NMR spectra of **SrPd-5**, **SrPd-6**, and *β*-CD (20 mM for ^{1}H and 100 mM for ^{13}C, pH = 7.0, D_2O, 298K).

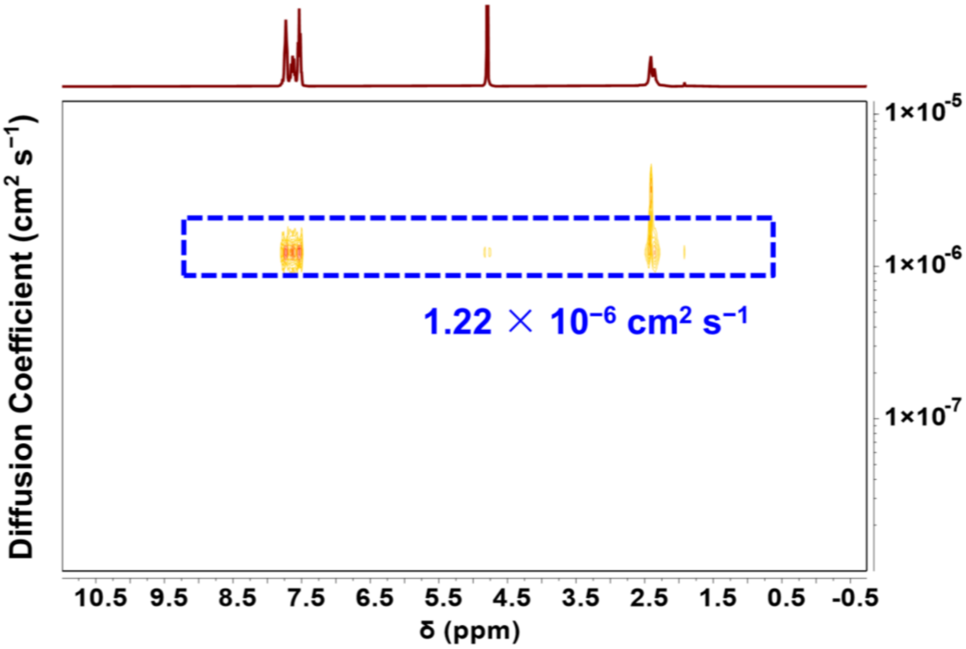

Fig. S11. 2D ^{1}H DOSY NMR spectrum of **SrPd-4** (60 mM, pH = 7.0, D_2O, 298K).

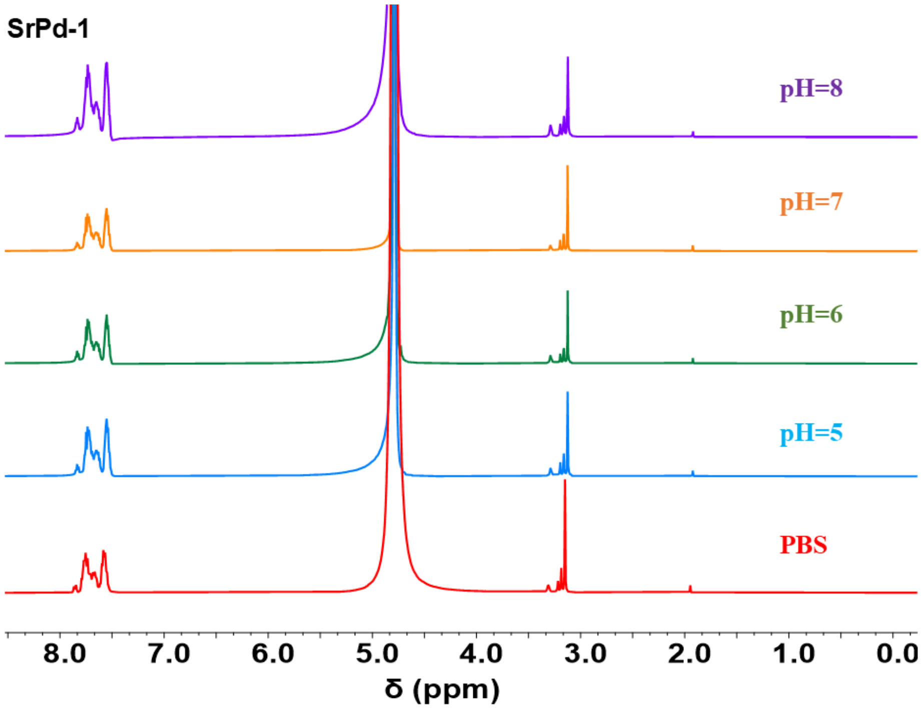

Fig. S12. ^{1}H NMR spectra of **SrPd-1** dissolved in D_2O at pH = 5.0-8.0 and PBS buffer (pH = 7.4) (20 mM, 298K).

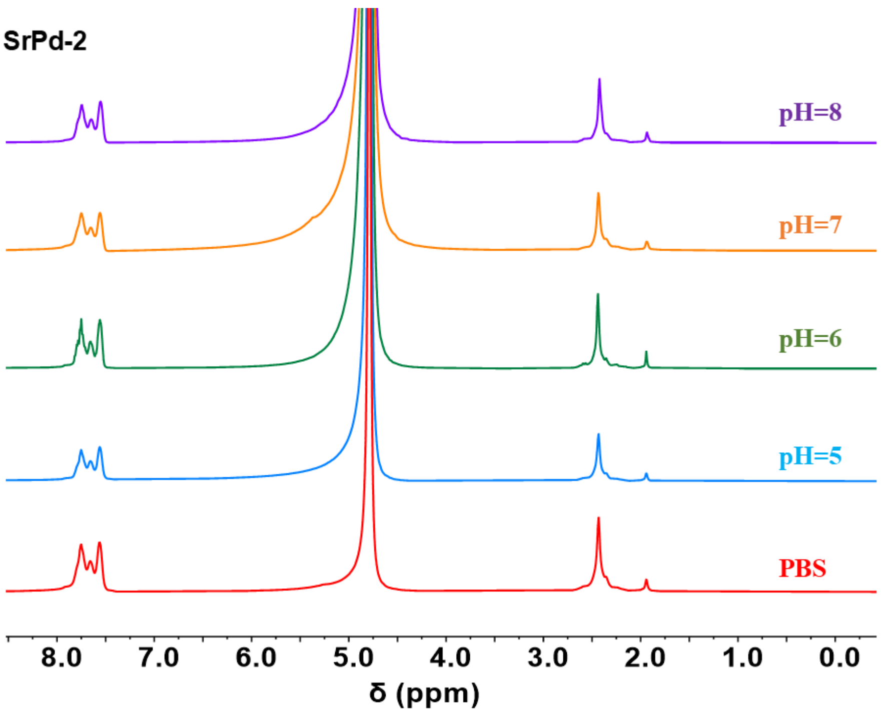

Fig. S13. ^{1}H NMR spectra of **SrPd-2** dissolved in D_2O at pH = 5.0-8.0 and PBS buffer (pH = 7.4) (20 mM, 298K).

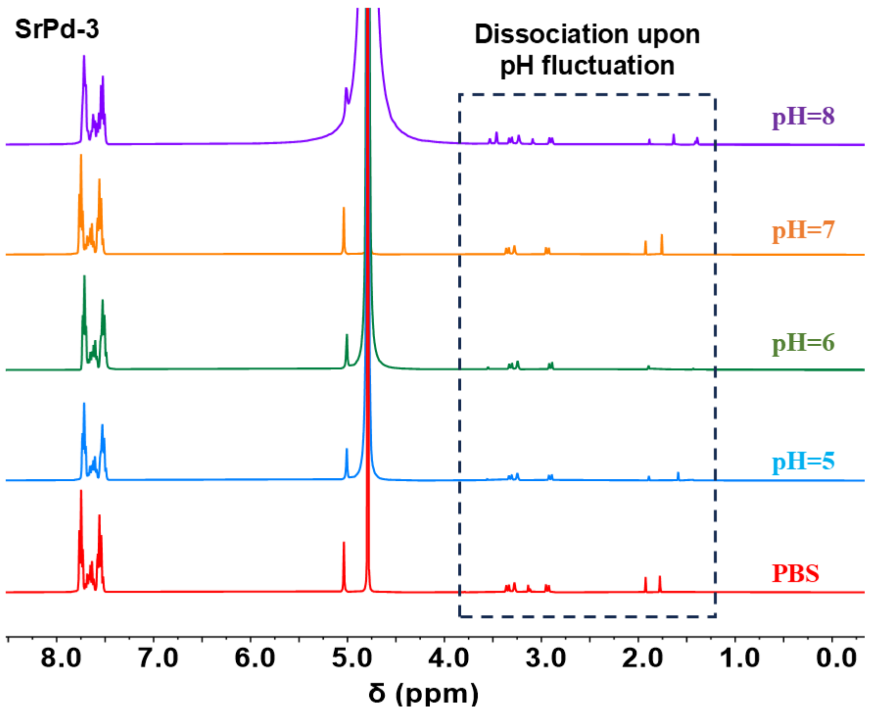

Fig. S14. ^{1}H NMR spectra of **SrPd-3** dissolved in D_2O at pH = 5.0-8.0 and PBS buffer (pH = 7.4) (20 mM, 298K).

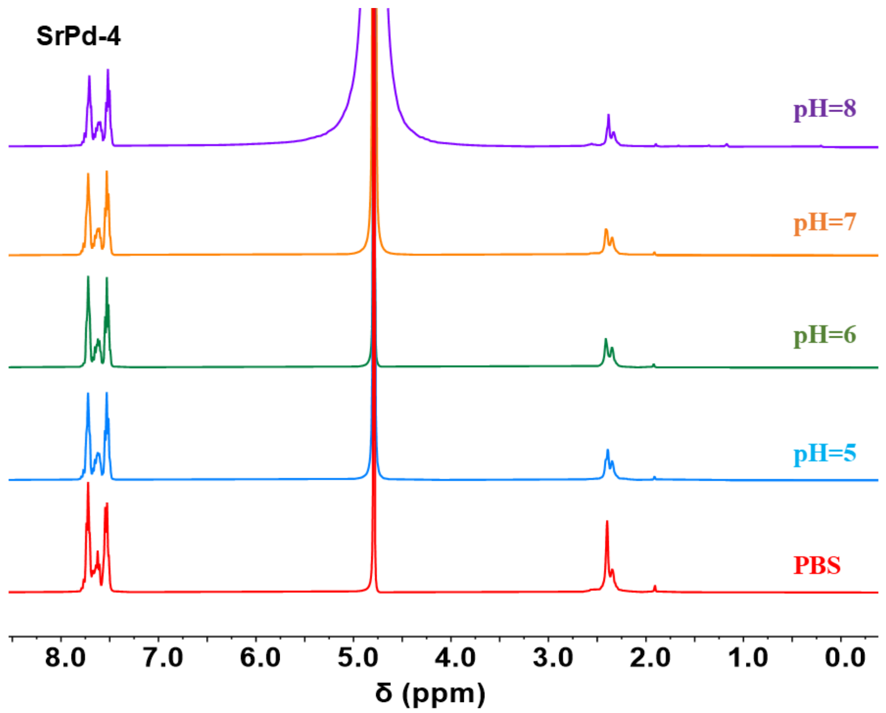

Fig. S15. ^{1}H NMR spectra of **SrPd-4** dissolved in D_2O at pH = 5.0-8.0 and PBS buffer (pH = 7.4) (20 mM, 298K).

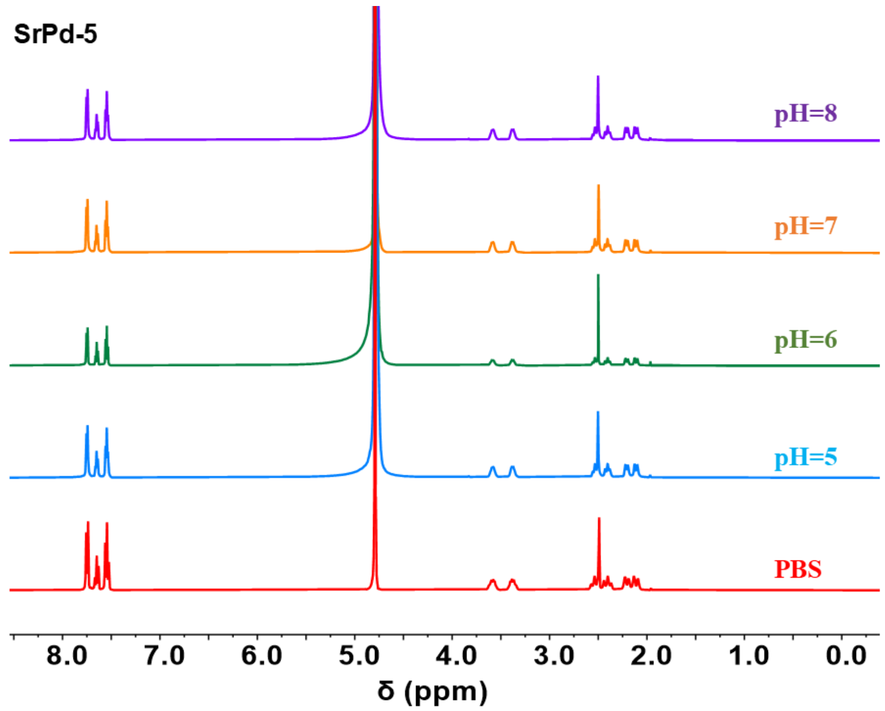

Fig. S16. ^{1}H NMR spectra of **SrPd-5** dissolved in D_2O at pH = 5.0-8.0 and PBS buffer (pH = 7.4) (20 mM, 298K).

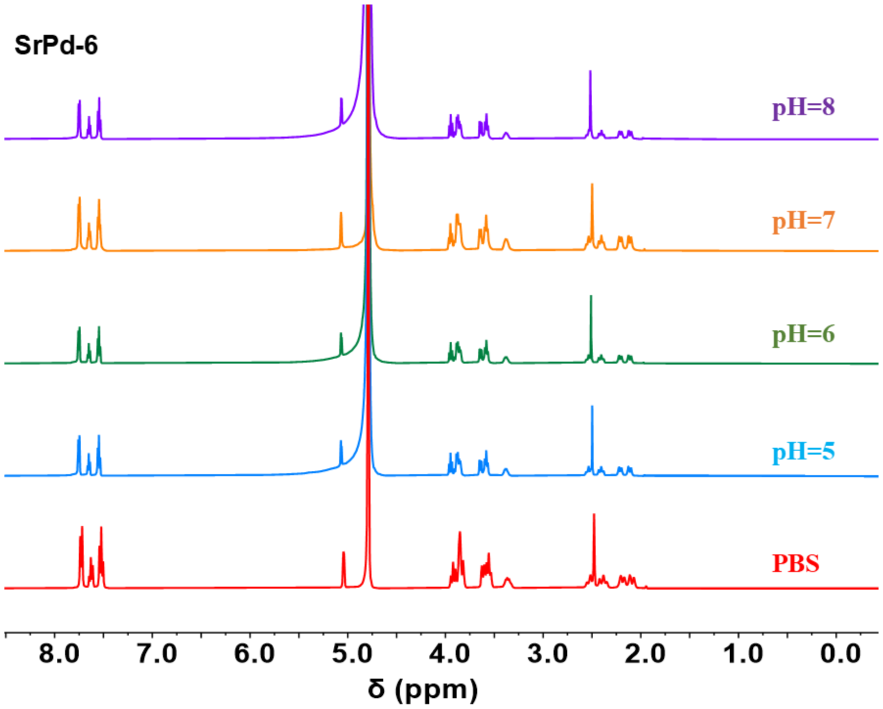

Fig. S17. ^{1}H NMR spectra of **SrPd-6** dissolved in D_2O at pH = 5.0-8.0 and PBS buffer (pH = 7.4) (20 mM, 298K).

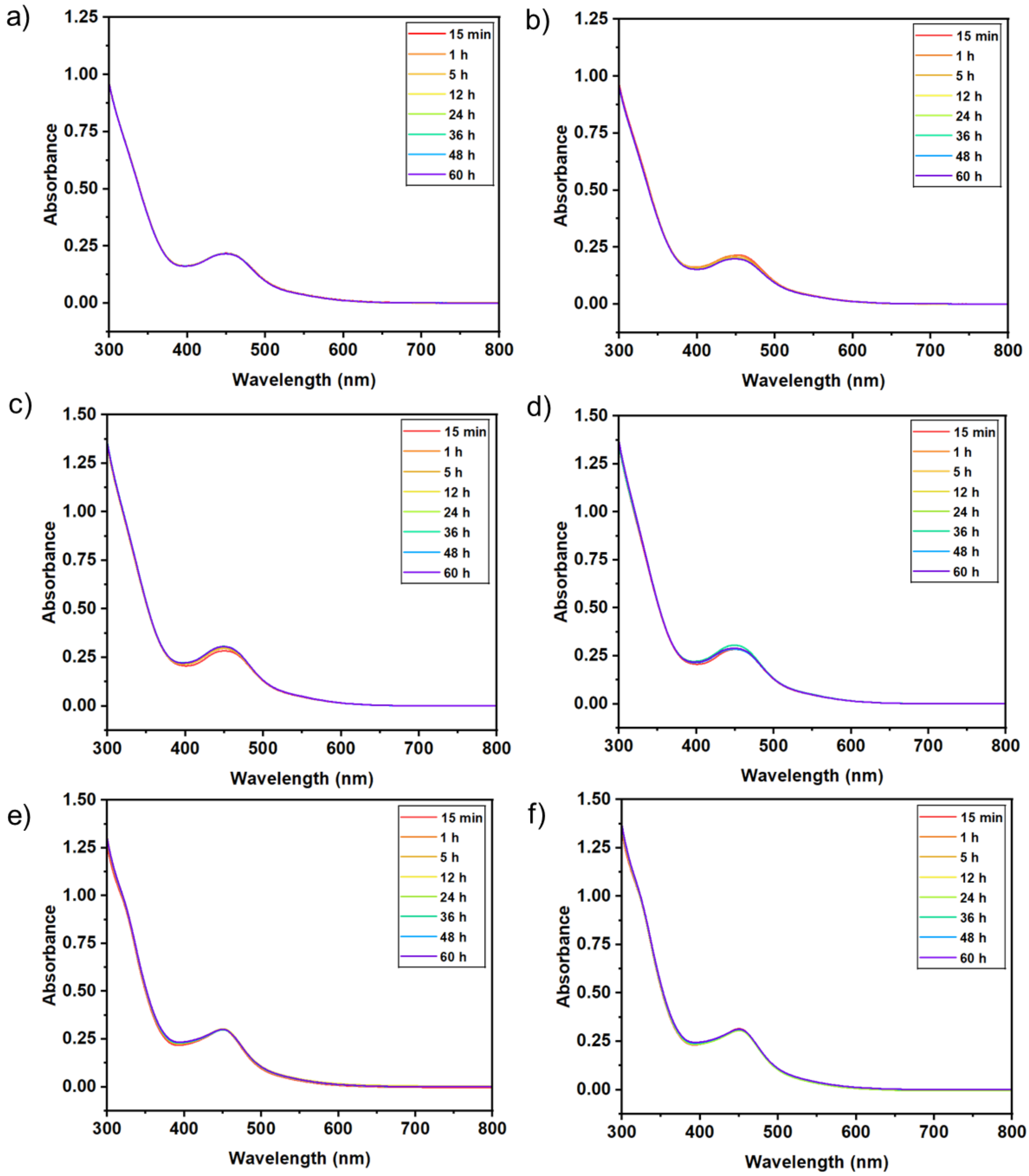

Fig. S18. UV-vis spectra of **SrPd-1** (a), **SrPd-2** (b), **SrPd-3** (c), **SrPd-4** (d), **SrPd-5** (e) and **SrPd-6** (f) in H_2O. The concentration of POPs was 2.0×10^{-6} M.

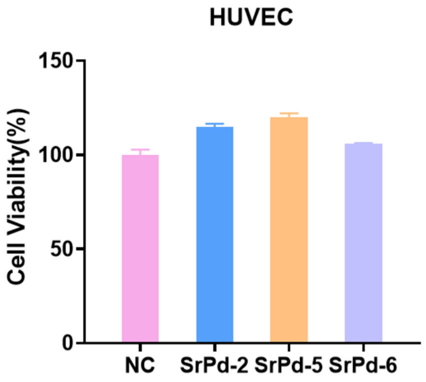

Fig. S19. CCK-8 assay on HUVEC cells treated with **SrPd-2**, **SrPd-5** and **SrPd-6** (n=3, mean ± SD).

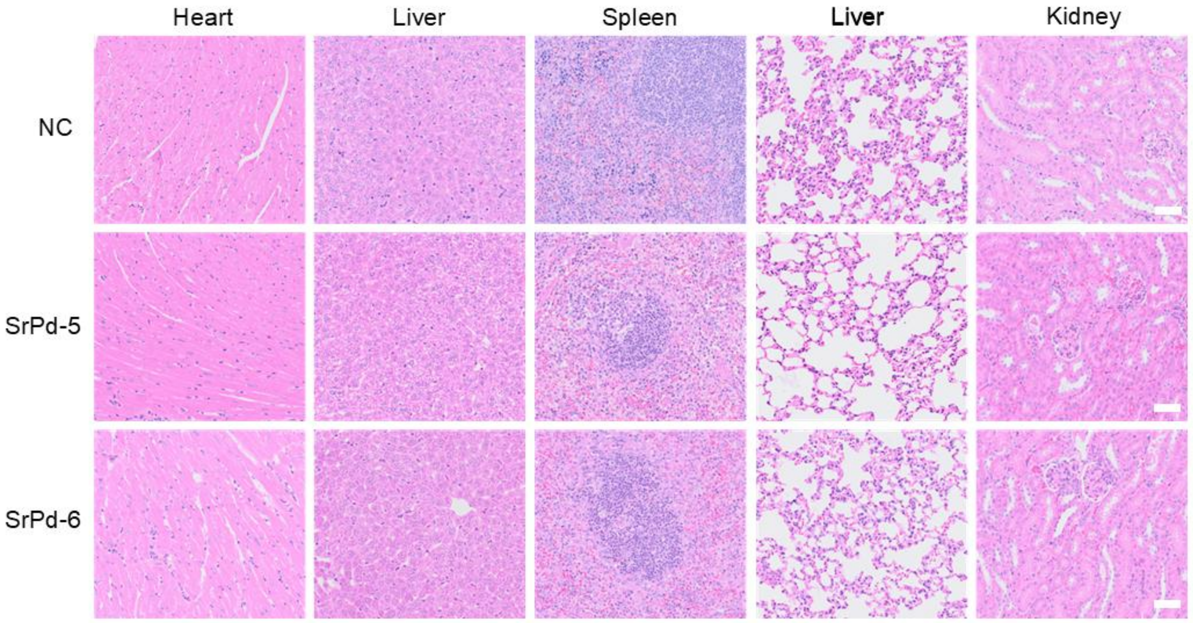

Fig. S20. Safety evaluation of **SrPd-5** and **SrPd-6** in major organs. Representative H&E staining images of major organs (heart, liver, spleen, lung, and kidney) harvested from mice in different treatment groups (NC, **SrPd-5**, and **SrPd-6**) at the end of the study period. Scale bar: 20 μm.

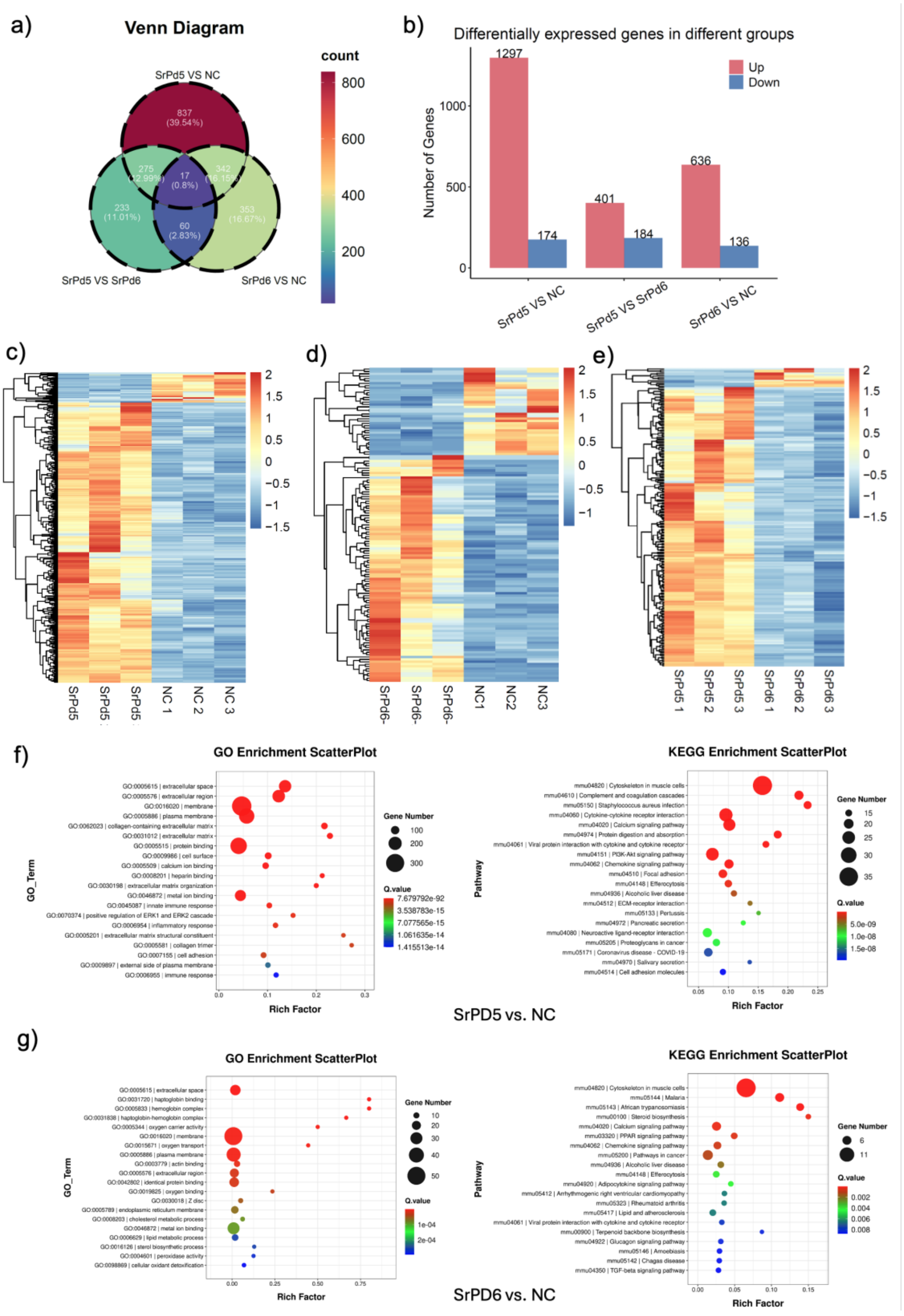

Fig. S21. Transcriptomic analysis of tumor samples from different treatment groups. (a) Venn diagram of differentially expressed genes (DEGs) overlapping among **SrPd-5** vs NC, **SrPd-6** vs NC, and **SrPd-5** vs **SrPd-6** comparisons. (b) Bar plot of upregulated and downregulated DEG counts across the comparison groups. (c-e) Hierarchical clustering heatmaps of DEGs between **SrPd-5** and NC (c), **SrPd-6** and NC (d), and **SrPd-5** vs **SrPd-6** (e). (f, g) GO and KEGG enrichment analysis of DEGs in **SrPd-5** vs NC (f) and **SrPd-6** vs NC (g).

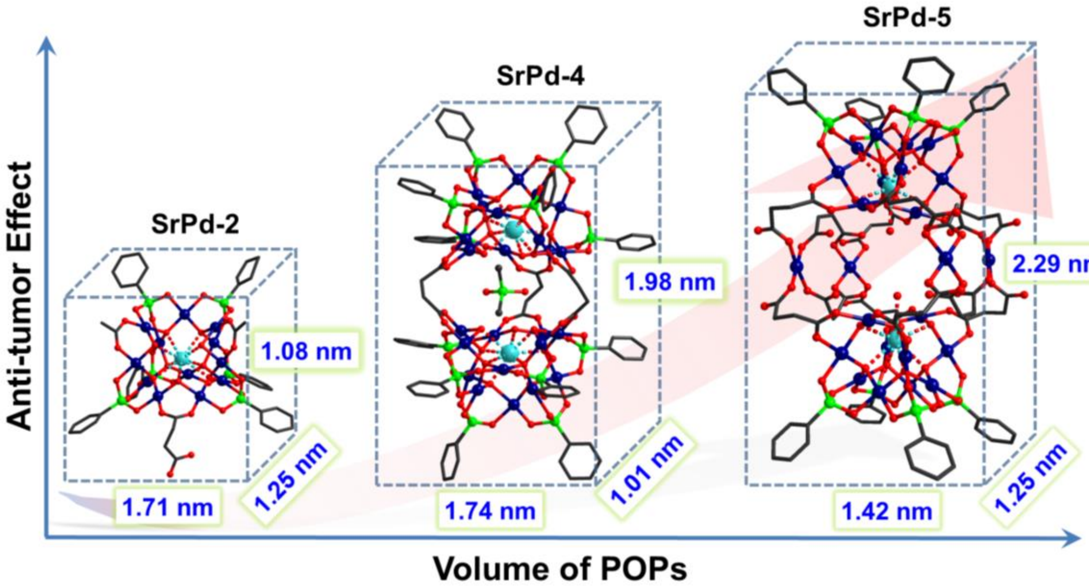

Fig. S22. Possible volume effect of as-made POPs on anti-tumor activity.

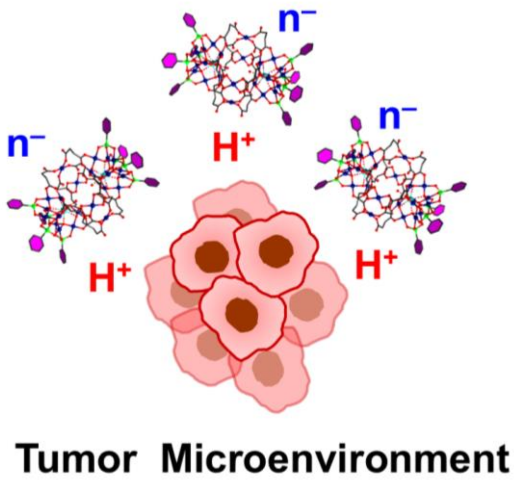

Fig. S23. Electrostatic attraction of POPs within the acidic tumor microenvironment.

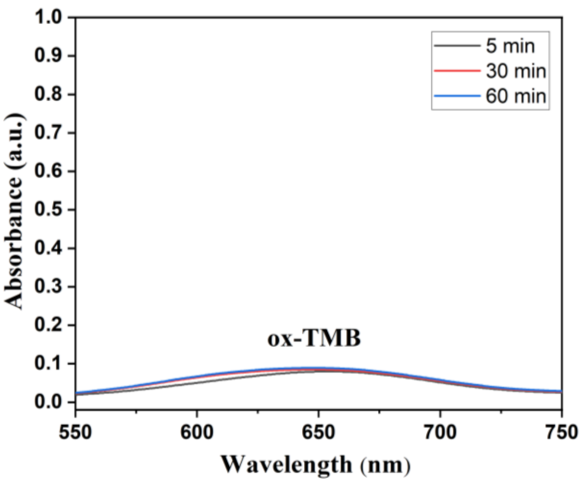

Fig. S24. UV–vis absorbance of ox-TMB in the presence of **SrPd-5** at different time intervals. The oxidation of TMB → ox-TMB has been used to determine the concentration of hydroxyl radical as oxidant, which is produced via the catalytic conversion of H_2O_2 by POPs. The limited absorbance at 652 nm corresponds to the low concentration of ox-TMB, indicating the poor production capacity of hydroxyl radical by **SrPd-5** as catalyst. (reaction condition: **SrPd-5** (20 μL, 4 mg·mL^{-1}), H_2O_2 (20 μL, 3 mM), and TMB (20 μL, 20 mM) dissolved in PBS buffer (2 mL, 0.01 M, pH 7.4).

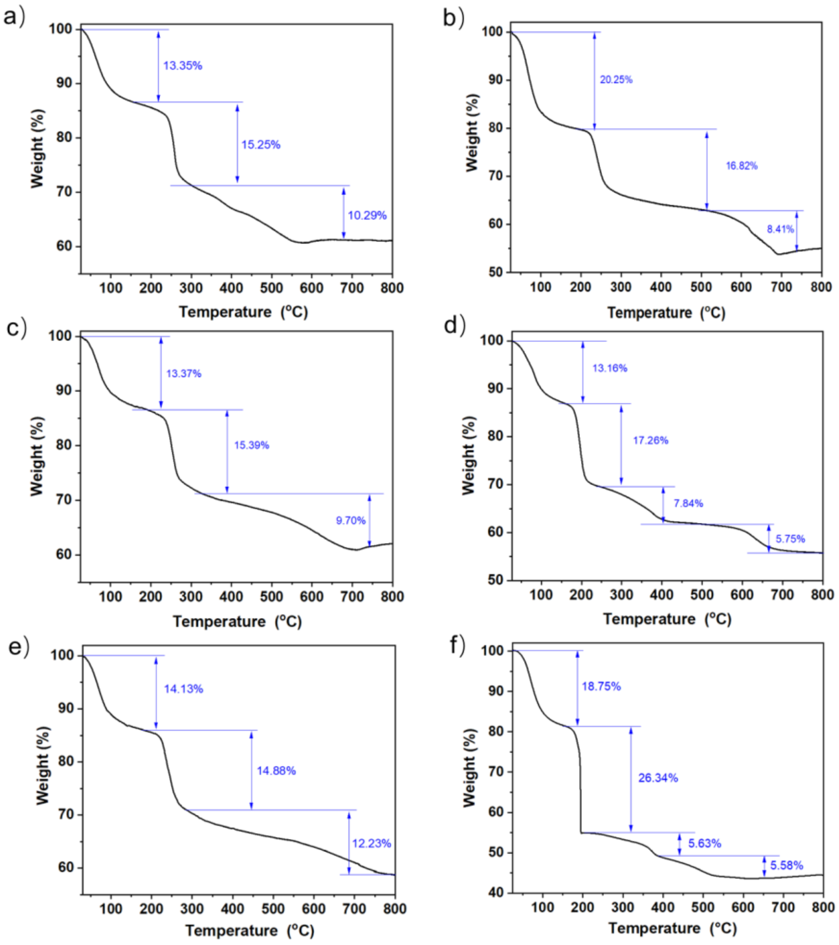

Fig. S25. Thermogram of **SrPd-1** (a), **SrPd-2** (b), **SrPd-3** (c), **SrPd-4** (d), **SrPd-5** (e) and **SrPd-6** (f) from 25 to 800 °C under N_2 atmosphere.

Table S1. Assignments and *m*/*z* values for the main peaks observed in the ESI-MS spectra.

m*/*z	**Formula**
	**SrPd-1**
984.72	$[H_2SrPd_{12}O_6(OH)_3(C_6H_5AsO_3)_6(C_3H_2O_4)_2(H_2O)_2]^{3-}$
992.06	$[NaHSrPd_{12}O_6(OH)_3(C_6H_5AsO_3)_6(C_3H_2O_4)_2(H_2O)_2]^{3-}$
1007.39	$\{H_4$**SrPd-1**$\}^{3-}$
	**SrPd-3**
1174.05	$\{H_4$**SrPd-3**$\}^{5-}$
1181.24	$\{H_4$**SrPd-3**$(H_2O)_2\}^{5-}$
1189.04	$\{KH_3$**SrPd-3**$(H_2O)_2\}^{5-}$
1196.63	$\{K_2H_2$**SrPd-3**$(H_2O)_2\}^{5-}$
1204.22	$\{K_3H$**SrPd-3**$(H_2O)_2\}^{5-}$
	**SrPd-4**
1182.45	$\{H_4$**SrPd-4**$\}^{5-}$
1189.65	$\{H_4$**SrPd-4**$(H_2O)_2\}^{5-}$
1197.44	$\{KH_3$**SrPd-4**$(H_2O)_2\}^{5-}$
1205.03	$\{K_2H_2$**SrPd-4**$(H_2O)_2\}^{5-}$
	**SrPd-5**
1516.72	$\{H_8$**SrPd-5**$(H_2O)_2\}^{4-}$
1526.21	$\{KH_7$**SrPd-5**$(H_2O)_2\}^{4-}$
1555.23	$\{K_5H_3$**SrPd-5**$\}^{4-}$
1564.97	$\{K_6H_2$**SrPd-5**$\}^{4-}$
1574.45	$\{K_7H$**SrPd-5**$\}^{4-}$

Table S2. BVS values for different structural types of oxygen in **SrPd-1**/**2**/**3**/**4**/**5**.

SrPd-1			
**μ_4-O (Sr, 3Pd)**	**BVS value**	**μ_2-O (As, Pd)**	**BVS value**
O2S	2.045	O1	1.803
O4S	2.059	O2	1.783
O5S	2.066	O3	1.773
O6S	2.062	O6	1.797
O7S	2.042	O7	1.822
O9S	2.053	O8	1.725
**μ_3-O (Sr, 2Pd)**	**BVS value**	O11	1.821
O1S	1.094	O12	1.807
O3S	1.123	O33	1.839
O8S	1.073	O14	1.805
**μ_2-O (C, Pd)**	**BVS value**	O15	1.743
O4	2.017	O16	1.739
O5	1.886	O19	1.848
O9	1.773	O20	1.719
O10	1.872	O21	1.776
O17	1.995	O22	1.837
O18	1.887	O23	1.808
		O28	1.871
**SrPd-2**			
**μ_4-O (Sr, 3Pd)**	**BVS value**	**μ_2-O (As, Pd)**	**BVS value**
O1S	2.073	O1	1.803
O3S	2.061	O2	1.685
O4S	2.083	O3	1.797
O5S	2.032	O4	1.772
O6S	2.080	O5	1.804
O7S	2.037	O6	1.783
**μ_3-O (Sr, 2Pd)**	**BVS value**	O7	1.831
O2S	1.163	O11	1.780
O8S	1.061	O12	1.701
O9S	1.090	O13	1.837
**μ_2-O (C, Pd)**	**BVS value**	O14	1.839
O8	1.965	O15	1.752
O9	1.874	O16	1.831
O10	1.940	O17	1.803
O22	1.962	O18	1.889
O23	1.966	O19	1.858
O24	1.984	O20	1.775
		O21	1.840

SrPd-3			
**μ_4-O (Sr, 3Pd)**	**BVS value**	**μ_2-O (As, Pd)**	**BVS value**
O2S	2.065	O8	1.790
O3S	2.046	O9	1.709
O5S	2.077	O10	1.774
O6S	2.045	O11	1.786
O7S	2.020	O12	1.787
O8S	2.080	O13	1.767
**μ_3-O (Sr, 2Pd)**	**BVS value**	O14	1.790
O1S	1.141	O15	1.752
O4S	1.154	O16	1.763
O9S	1.152	O17	1.788
**μ_2-O (C, Pd)**	**BVS value**	O18	1.852
O1	1.910	O19	1.775
O2	1.955	O20	1.795
O3	1.938	O21	1.820
O4	2.040	O22	1.862
O5	2.042	O23	1.833
O6	1.978	O24	1.914
		O25	1.802
**SrPd-4**			
**μ_4-O (Sr, 3Pd)**	**BVS value**	**μ_2-O (C, Pd)**	**BVS value**
O18	2.026	O27	1.925
O19	2.045	O28	1.957
O20	2.055	O29	2.004
O21	2.013	O30	1.843
O23	2.034	O31	1.922
O26	2.016	O32	1.991
O57	1.993	O33	1.880
O58	1.983	O34	1.973
O59	1.933	O38	1.959
O60	1.930	O39	1.761
O62	2.013	O40	1.923
O64	2.012	O41	2.018
**μ_3-O (Sr, 2Pd)**	**BVS value**	**μ_2-O (As, Pd)**	**BVS value**
O22	1.127	O1	1.794
O24	1.013	O2	1.776
O25	1.133	O3	1.794
O61	1.109	O4	1.813
O63	1.118	O5	1.753
O65	1.090	O6	1.795

μ_2-O (As, Pd)	BVS value	μ_2-O (As, Pd)	BVS value
O7	1.745	O43	1.758
O8	1.761	O44	1.776
O9	1.890	O45	1.784
O10	1.787	O46	1.702
O11	1.776	O47	1.622
O12	1.738	O48	1.826
O13	1.847	O49	1.663
O14	1.692	O50	1.812
O15	1.758	O51	1.783
O16	1.806	O52	1.616
O17	1.870	O53	1.650
O35	1.790	O54	1.711
O36	1.727	O55	1.937
O37	1.573	O56	1.727
O42	1.670	O66	1.799
**SrPd-5**			
**μ_4-O (Sr, 3Pd)**	**BVS value**	O22	1.781
O1S	2.079	O23	1.768
O2S	2.045	**μ_2-O (C, Pd)**	**BVS value**
O3S	2.047	O2	1.814
O4S	2.095	O3	2.017
**μ_3-O (Sr, 2Pd)**	**BVS value**	O4	1.790
O5S	1.180	O5	2.086
O6S	1.192	O8	1.971
O7S	1.162	O9	1.932
O8S	1.169	O14	1.968
**μ_2-O (As, Pd)**	**BVS value**	O15	1.933
O6	1.859	O16	2.022
O7	1.795	O17	1.860
O10	1.720	O24	2.158
O11	1.733	O25	1.687
O12	1.743	O26	2.003
O13	1.768	O27	2.343
O18	1.754	O28	1.764
O19	1.751	O33	1.886
O20	1.725	**Terminal O (Sr)**	**BVS value**
O21	1.791	O1	0.501

Table S3. Crystal data and structure refinement for the as-made compounds.

Compound	**Na-SrPd-1**	**KNa-SrPd-2**	**KNa-SrPd-3**
Empirical formula	$Na_7SrPd_{12}As_6C_{45}O_{66}H_{93}$	$K_2Na_3SrPd_{12}As_6C_{44}O_{77}H_{127}$	$K_7Na_2Sr_2Pd_{24}As_{13}C_{83}O_{121}H_{184}$
Formula weight, g/mol	3665.30	3849.79	7141.24
Crystal system	Triclinic	Triclinic	Monoclinic
Space group	$P\bar{1}$	$P\bar{1}$	$C2/m$
a, Å	17.691(5)	18.071(3)	31.318(12)
b, Å	19.764(6)	18.587(3)	36.803(12)
c, Å	20.952(6)	19.091(3)	23.001(7)
α, °	63.941(13)	87.069(5)	90
β, °	71.272(16)	67.324(5)	107.973(10)
γ, °	76.975(17)	88.711(5)	90
Volume, $Å^3$	6201.8(3)	5909.2(15)	25216.8(15)
Z	2	2	8
D_{calc}, g/cm^3	1.766	1.828	1.684
Absorption coefficient, mm^{-1}	3.794	4.025	3.973
$F(000)$	3088	3056	11934
Theta range for data collection, °	2.362 to 28.424	2.162 to 25.000	2.166 to 25.000
Completeness to Θ_{max}	98.8%	99.8%	99.8%
Index ranges	-23<=h<=23<br>-26<=k<=26<br>-27<=l<=28	-21<=h<=21<br>-22<=k<=22<br>-22<=l<=22	-37<=h<=37<br>-43<=k<=41<br>-27<=l<=27
Reflections collected	195709	131122	112300
Independent reflections	30765	20800	22578
R(int)	0.0702	0.0762	0.2223
Absorption correction	Semi-empirical from equivalents	Semi-empirical from equivalents	Semi-empirical from equivalents
Data / restraints / parameters	30765 / 1944 / 1060	20800 / 1986 / 1047	22578 / 1992 / 970
Goodness-of-fit on F^2	1.048	1.079	1.052
R_1,[a] wR_2[b] ($I > 2\sigma(I)$)	R_1 = 0.0601<br>wR_2 = 0.1763	R_1 = 0.0480<br>wR_2 = 0.1345	R_1 = 0.0621<br>wR_2 = 0.1688
R_1,[a] wR_2[b] (all data)	R_1 = 0.0991<br>wR_2 = 0.2036	R_1 = 0.0609<br>wR_2 = 0.1400	R_1 = 0.0836<br>wR_2 = 0.1850
Largest diff. peak and hole, e/$Å^3$	1.938 and -1.237	1.909 and -1.591	1.986 and -1.506

[a] $R_1 = \Sigma||F_o| - |F_c||/\Sigma|F_o|$. [b] $wR_2 = [\Sigma w(F_o^2 - F_c^2)^2/\Sigma w(F_o^2)^2]^{1/2}$.

Compound	**KNa-SrPd-4**	**K-SrPd-5**	**K-SrPd-6**
Empirical formula	$K_7Na_3Sr_2Pd_{24}As_{13}C_{88}O_{125}H_{196}$	$K_{13}Sr_2Pd_{24}As_8C_{98}O_{151}H_{220}$	$K_{13}Sr_2Pd_{24}As_8C_{140}O_{215}H_{348}$
Formula weight, g/mol	7300.38	7651.68	9309.10
Crystal system	Triclinic	Monoclinic	Triclinic
Space group	*P*1̄	$P2_1/c$	*P*1̄
a, Å	16.429(15)	21.438(9)	16.735(6)
b, Å	21.460(18)	28.002(11)	17.409(5)
c, Å	32.193(3)	21.057(8)	30.234(8)
α, °	102.607(3)	90	75.238(10)
β, °	101.219(3)	104.119(2)	76.008(10)
γ, °	106.307(3)	90	72.312(10)
Volume, Å^3	10226.7(15)	12258.8(9)	7984.5(4)
Z	2	4	1
D_{calc}, g/cm^3	2.042	1.734	1.551
Absorption coefficient, mm^{-1}	4.846	3.369	2.597
F(000)	5869	6046	3574
Theta range for data collection, °	2.147 to 25.000	2.125 to 25.000	2.139 to 25.000
Completeness to Θ_{max}	99.9%	98.5%	99.9%
Index ranges	-19<=h<=19<br>-25<=k<=25<br>-38<=l<=38	-25<=h<=25<br>-31<=k<=33<br>-25<=l<=25	-19<=h<=19<br>-20<=k<=20<br>-35<=l<=35
Reflections collected	178246	155966	140476
Independent reflections	36012	21253	55761
R(int)	0.1591	0.0877	0.0335
Absorption correction	Semi-empirical from equivalents	Semi-empirical from equivalents	Semi-empirical from equivalents
Data / restraints / parameters	36012 / 5688 / 2149	21253 / 2733 / 1153	55761 / 7791 / 2750
Goodness-of-fit on F^2	1.030	1.078	1.036
R_1,[a] wR_2[b] (I> 2σ(I))	R_1 = 0.0721<br>wR_2 = 0.1933	R_1 = 0.0676<br>wR_2 = 0.1782	R_1 = 0.0387<br>wR_2 = 0.0985
R_1,[a] wR_2[b] (all data)	R_1 = 0.1106<br>wR_2 = 0.2290	R_1 = 0.0897<br>wR_2 = 0.1920	R_1 = 0.0464<br>wR_2 = 0.1033
Largest diff. peak and hole, e/Å^3	2.439 and -1.729	2.403 and -1.218	1.030 and -0.647

[a] $R_1 = \Sigma||F_o| - |F_c||/\Sigma|F_o|$. [b] $wR_2 = [\Sigma w(F_o^2 - F_c^2)^2/\Sigma w(F_o^2)^2]^{1/2}$.

References

[S1] P. Yang, Y. Xiang, Z. Lin, B. S. Bassil, J. Cao, L. Fan, Y. Fan, M.-X. Li, P. Jiménez-Lozano, J. J. Carbó, J. M. Poblet, U. Kortz, *Angew. Chem. Int. Ed.* **2014**, *53*, 11974–11978.

[S2] G. M. Sheldrick, *Acta Cryst.* **2015**, *C71*, 3–8.

[S3] G. M. Sheldrick, *Acta Cryst.* **2008**, *A64*, 112–122.

[S4] O. V. Dolomanov, L. J. Bourhis, R. J. Gildea, J. A. K. Howard, H. Puschmann, *J. Appl. Cryst.* **2009**, *42*, 339–341.

[S5] A. L. Spek, *Acta Cryst.*, **2015**, *C71*, 9–18.
